# 16S rRNA and metabolomics reveal the key microbes and key metabolites that regulate diarrhea in Holstein male calves

**DOI:** 10.3389/fmicb.2024.1521719

**Published:** 2025-01-15

**Authors:** P. P. Cao, C. L. Hu, M. J. Li, Y. H. An, X. Feng, X. H. Ma, D. Z. Wang, Z. H. Song, G. S. Ji, D. Yang, Q. Ma, W. F. Yang, J. N. Dong, H. R. Zhang, Y. Ma, Y. F. Ma

**Affiliations:** ^1^College of Animal Science and Technology, Ningxia University, Yinchuan, China; ^2^Ningxia Borui Technology Co., Ltd, Yinchuan, China; ^3^Ningxia Xin'ao Agriculture and Animal Husbandry Co., Ltd, Yinchuan, China

**Keywords:** diarrhea, Holstein male calves, 16S rRNA, untargeted metabolomics, growth performance

## Abstract

**Introduction:**

Diarrhea is a prevalent disease among calves, which significantly hinders their growth and development, thereby impacting farm productivity and revenue. This study aimed to investigate the impact of diarrhea on calf growth.

**Methods:**

Holstein male calves with similar birth weight (39.5 ± 4.2 kg) were included in this study, and key parameters such as fecal score, diarrhea incidence, and growth performance from birth to weaning were measured. Rectal fecal samples from both diarrheic (*n* = 24) and healthy calves (*n* = 24) aged 1–4 weeks were analyzed using 16S rRNA gene sequencing and untargeted metabolomics.

**Results:**

Our findings indicated a high prevalence of diarrhea among calves between 1–4 weeks of age on pasture, which led to a marked decrease in growth performance, including average daily gain. At the genus level, the relative abundance of *GCA-900066575* in one-week-old diarrheic calves was significantly higher; *Escherichia-Shigella* and *Pseudoflavonifractor* were more abundant in two-week-old calves; while *Tyzzerella* and *Lachnospiraceae_UCG-004* increased significantly in four-week-old calves, and correlated negatively with average daily gain, suggesting that these bacteria may promote the occurrence of diarrhea. Correlation analysis revealed that fecal metabolites such as arachidonic acid, cis-vaccenic acid, oleic acid, choline, creatinine, and others were significantly negatively correlated with calf growth performance and were significantly increased in diarrheic calves. WGNCA identified that dark magenta module metabolites were significantly associated with diarrhea traits from 1–4 weeks. Thirteen metabolites, including glycerophospholipids (such as 1-stearoyl-2-hydroxy-sn-glycero-3-phosphoethanolamine), fatty acids (such as dodecanoic acid), and arachidonic acid, were positively correlated with *GCA-900066575*, *Escherichia-shigella*, *Tyzzerella*, and *Clostridium_butyricum*, but negatively correlated with *UBA1819*, *Lachnoclostridium_sp_YL32*, and *Clostridium_scindens*.

**Discussion:**

Therefore, *GCA-900066575*, *Escherichia-shigella*, *Lachnospiraceae_UCG-004*, and *Tyzzerella* are likely key bacterial genera causing diarrhea in calves, while arachidonic acid, glycerol phospholipids, and fatty acids are critical metabolites associated with this condition. These alterations in the fecal microbiota and metabolite composition were found to be the principal contributors to growth retardation in diarrheic calves.

## 1 Introduction

Neonatal calf diarrhea remains a predominant cause of morbidity and mortality, contributing to over 50% of total calf deaths (Cho and Yoon, 2014). This condition severely compromises calf welfare and incurs substantial economic losses within the cattle industry (Pempek et al., 2019). The primary etiological factor is infection by intestinal pathogens, attributed to the immature development of the calf’s gut and its vulnerability to pathogen invasion, which might lead to diarrhea (Cho and Yoon, 2014). Pathogen proliferation in the gastrointestinal tract diminishes beneficial bacteria populations, with pathogens such as *Escherichia coli*, bovine rotavirus, bovine coronavirus, and *Cryptosporidium* spp. being closely implicated in neonatal calf diarrhea (Brunauer et al., 2021). Intestinal damage further exacerbates permeability, allowing pathogens to breach the intestinal immune barrier and trigger inflammatory response, ultimately impairing calf growth and development (Ying et al., 2013).

Recent studies have revealed the significance of integrating microbiome and metabolome data to elucidate host–microbe interactions and identify predictive biomarkers for disease (Visconti et al., 2019). Metabolites offer a comprehensive view of metabolic alterations in the gut, enhancing our understanding of the mechanisms by which intestinal dysbiosis precipitates the emergence of diarrhea. Amin et al. (2023) demonstrated a robust correlation between fecal microorganisms, plasma metabolites, and calf growth performance from 42 to 98 days postpartum.

Growing evidence has indicated a significant relationship between gut microbiota and animal health (Esser et al., 2019). For instance, diarrheic calves have been described to exhibit reduced *Bacteroides* levels and elevated *Proteobacteria* levels in their feces (Kim E. T. et al., 2021). Moreover, it is known that the gut microbiota of healthy calves promotes the maturation of the intestinal immune system by the production of beneficial metabolites such as short-chain fatty acids (SCFAs) (Shi et al., 2017). For instance, *Bacteroides* levels are linked to SCFA regulation (Horvath et al., 2022), which in turn stimulates bicarbonate secretion in the intestine (Dohgen et al., 1994). Shi et al. (2023) suggested that calf diarrhea might be associated with gut microbiota imbalance affecting acid–base homeostasis and highlighted the role of microbiota-driven disruptions in purine and arachidonic acid metabolism. Moreover, infection with bovine rotavirus and coronavirus has been shown to alter fecal microbiota composition and metabolites (Cui et al., 2023). Therefore, targeted modulation of the gut microbiota constitutes a potential strategy for preventing and treating calf diarrhea (Shi et al., 2023). However, despite the established link between altered gut microbiota and diarrhea, the microbial and metabolic mechanisms connecting diarrhea to hindered growth and development in calves remain poorly understood.

In the present study, using 16S rRNA gene sequencing and non-targeted metabolomics, differences in fecal metabolites and microbiota between healthy and diarrheic calves aged 1–4 weeks were investigated. The relationship between changes in the intestinal microbiota and metabolites and growth retardation in diarrheic calves was investigated.

## 2 Materials and methods

### 2.1 Animals

Calves included in the present study were sourced from a large-scale dairy farm in Lingwu, Ningxia, China. Sixty one-day-old Holstein male calves showing similar body weight at birth (39.5 ± 4.2 kg, *n* = 60) were selected and managed according to the farm’s standard feeding protocols. Throughout the trail period, which lasted from birth to weaning for 60 days, calves were isolated from other cows and housed individually on calf islands. No treatments were administered other than routine umbilical cleaning.

Within 6 h of birth, each calf received 2.0 L of colostrum (2.0 L after 2 h and 2.0 L after 4–6 h of birth). On the second day, calves were moved to calf islands and fed regular milk (EKOMILK Bond, Europe) whose composition is detailed in Supplementary Table S1 at a rate of 4 L/day in two feedings. By day 30, daily milk intake was gradually increased to 6 L/day and maintained at this level until weaning on day 60. Clean, fresh water was available from day 3, and hay and calf concentrate supplements (COFCO Feeds, Yinchuan Plant Co., Ltd.) (Supplementary Table S2) were provided from day 7. Fecal scores, color, and status were recorded using a three-point scale (0 = normal feces; 1 = semi-formed feces; 2 = loose feces; 3 = watery feces) at the beginning of the experiment. A diarrhea group and a healthy group were formed to monitor diarrhea incidence calculated based on the below formula.


$$\mathrm{diarrhea}\;\mathrm{rate}\;\left(\%\right)\;=\;\frac{\begin{array}{l} \mathrm{number of calves with}\;\\ \mathrm{diarrhea}\times \mathrm{diarrhea days} \end{array}}{\begin{array}{l} \mathrm{the total number of}\;\\ \mathrm{calves}\times \mathrm{experimental days} \end{array}}\times 100\%$$

Feed intake, body length, and size were recorded from birth to weaning (see Supplementary Table S3 for measurement indicators). Calves that were medicated, had other diseases and died during the trial were eliminated.

### 2.2 Experimental design, and sample collection

**Figure 1:**
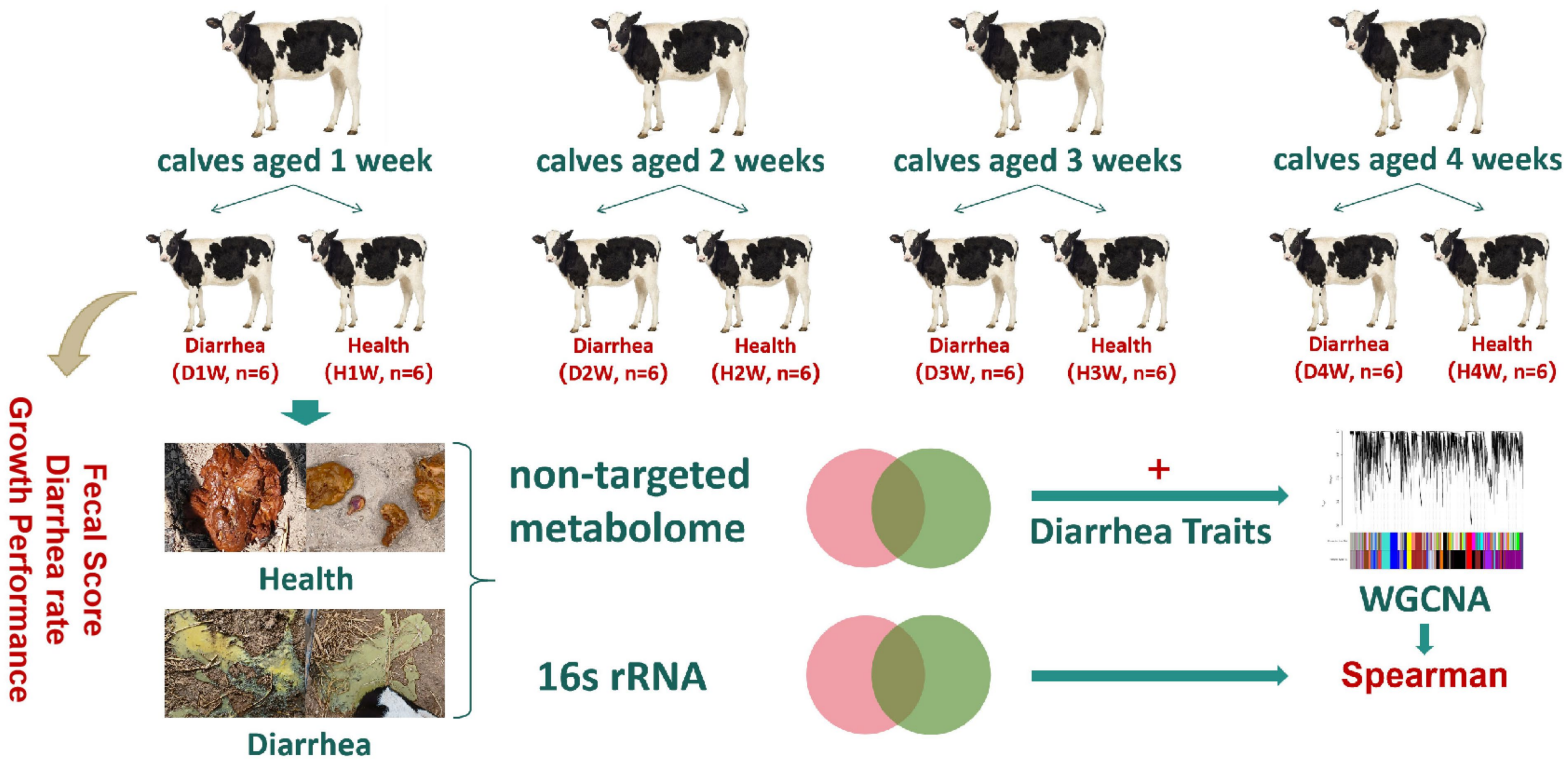
Experimental design.

The study was divided into periods of 4 weeks (week 1, week 2, week 3, and week 4) and based on diarrhea incidence. Each week calves were grouped into diarrhea and healthy groups: (i) D1W (diarrhea during week 1, *n* = 6) and H1W (healthy during week 1, *n* = 6); (ii) D2W (diarrhea during week 2 and no diarrhea on week 1, *n* = 6), H2W (healthy during week 2, *n* = 6); (iii) D3W (diarrhea during week 3 and no diarrhea on weeks 1 and 2, *n* = 6) and H3W (healthy during week 3, *n* = 6); (iv) D4W (diarrhea during week 4 and no diarrhea on weeks 1 to 3, *n* = 6,) and H4W (healthy during week 4, *n* = 6). Fifty grams of fecal content were collected from both diarrheic (*n* = 24) and healthy calves (*n* = 24) by rectal stimulation using a sterile glove (Figure 1). Samples were placed in frozen storage tubes, quickly transferred to liquid nitrogen tanks, and stored at −80°C. The morphology of fecal samples was observed and recorded as described earlier. Supplementary Table S4 provides details on sample groups and collection methods.

### 2.3 Metabolomics analysis

Fecal samples collected from the 48 calves were analyzed using LS-MS untargeted metabolomics sequencing. All sample scans were performed on an LC–MS system according to the manufacturer’s instructions. Samples were separated by ultra-high performance liquid chromatography (Agilent 1290 Infinity LC, China) using a hydrophilic interaction liquid chromatography column. To mitigate the influence of fluctuations in the instrument detection signal, samples were analyzed continuously in random order. Quality control samples were included to monitor system stability and ensure reliability of experimental data.

A mass spectrometer (AB Triple TOF 6600, Basao, China) was used to collect primary and secondary spectra. We used a combination of both positive ion mode (POS) and negative ion mode (NEG) ionization modes in our experiments, which resulted in better metabolite coverage and detection. Data were analyzed using principal component analysis (PCA) (Warnes, 2007) and orthogonal partial least squares discriminant analysis (OPLS-DA) (Thevenot, 2016) using the R package gmodels. We combined multivariate statistical analysis of OPLS-DA for VIP values and univariate statistical analysis of *p*-values to screen for significantly differentially enriched metabolites between different comparison groups (Saccenti et al., 2014). Thresholds for significant differences were considered based on VIP values ≥1 and *p*-values <0.05 in the OPLS-DA model. Metabolic function and metabolic pathway analyses of differential metabolites were performed using the KEGG database. Untargeted metabolomics sequencing was carried out by Baseo, Guangzhou, China.

### 2.4 16s rRNA data analysis

Fecal samples from the 48 calves were analyzed using 16S rRNA gene sequencing. Microbial DNA was extracted using HiPure Soil DNA kits (or HiPure Stool DNA kits) (Magen, Guangzhou, China) according to the manufacturer’s instructions. Samples were stored at −80°C in a refrigerator until library preparation and sequencing could be performed. Amplification of the V3–V4 hypervariable region of the 16 s rRNA gene was performed using an Illumina sequencing platform with index-binding primer pairs 341F (5’-CCTACGGGGNGGCWGCAG-3′) and 806R (5’-GGACTACHVGGGTATCTAAT-3′) (Guo et al., 2017). Agarose gel electrophoresis was performed to verify amplicon sizes. Library quality was assessed using ABI StepOnePlus Real-Time PCR System (Life Technologies, Foster City, United States). Libraries were sequenced on a Novaseq 6000 platform generating 2 × 250 bp paired-end reads. Second-round amplification products were purified using AMPure XP Beads, quantified using the ABI StepOnePlus Real-Time PCR System (Life Technologies, United States), and sequenced on-line according to the PE250 pattern pooling of the Novaseq 6000 platform.

Four distance metrics were calculated based on operational taxonomic unit (OTU) abundance tables. For alpha- and beta-diversity analyses, OTU representative sequences were first aligned using Muscle (version 3.8.31) software (Edgar, 2004), and comparison files were imported into FastTree (version 2.1) software (Price et al., 2010) to construct a phylogenetic tree. Unweighted and weighted UniFrac distance analyses were then performed using the GuniFrac package (version 1.0) (Lozupone and Knight, 2005) in R. To assess alpha-diversity, indices were conducted using QIIME (version 1.9.1) software (Caporaso et al., 2010). Jaccard and Bray-Curtis distance matrices were calculated using R project Vegan package (version 2.5.3) (Oksanen, 2010). Functional annotation of bacterial KEGG pathways was performed using PICRUSt (version 2.1.4) software (Langille et al., 2013), and the abundance of information for each pathway was quantified. 16S sequencing was carried out by Baseo, Guangzhou, China.

### 2.5 WGCNA and KEGG enrichment analysis

WGCNA analysis was performed using the Kidio Cloud platform.^1^ Low-expression metabolites were filtered out before importing metabolite expression data into R. Co-expression modules were generated using default network construction settings, with a power value of 8 and a minimum module size of 50. The weighted co-expression network model was constructed with “diarrhea” and “health” as traits. KEGG enrichment analysis was applied to metabolites in each module to determine the biological functions associated with the identified co-expression clusters.

### 2.6 Statistical analysis

Spearman correlation coefficients between fecal microbial species, fecal metabolites, and growth performance traits were calculated using the R language psych package (version 1.8.4). Heatmaps were generated using the pheatmap package to visualize the relationships between fecal metabolomes and 16S rRNA profiles in diarrheic and healthy calves. Data were expressed as mean ± standard deviation, and Welch’s t-test or Wilcoxon rank sum test was used to compare phenotypic differences between groups. Growth performance data were analyzed for significance of differences by one-way ANOVA and t-tests using GraphPad Prism (version 9.3.0) software. All statistical tests were two-tailed, and significance was set at *p* < 0.05. * indicates *p* < 0.05 for significant difference, ** indicates *p* < 0.01, *** indicates *p* < 0.001, and **** indicates *p* < 0.0001 for highly significant difference.

## 3 Results

### 3.1 Diarrhea rate and growth performance of calves

**Figure 2:**
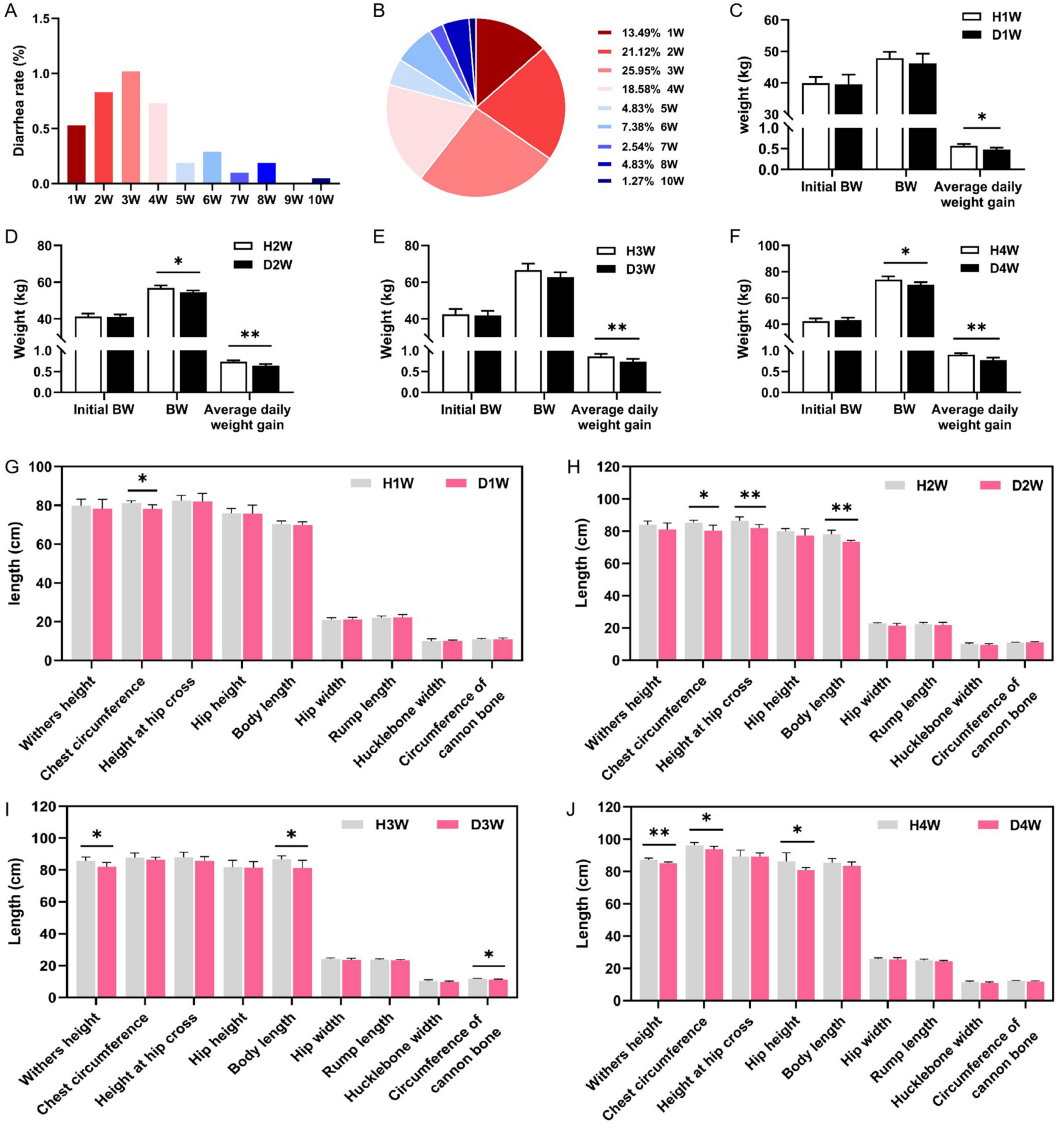
Diarrhea rate and growth performance of calves. Diarrhea rate of 1–10 w in newborn calves **(A)**. Statistical table of the pie charts **(B)**. Growth performance indices of D1W calves at 14 d **(C,G)**. Growth performance indices of D2W calves at 21 d **(D,H)**. Growth performance indices of D3W calves at 28 d **(E,I)**. Growth performance indices of D4W calves at 36 d **(F,J)**. *p < 0.05,**p < 0.01.

Body weight and various growth performance metrics of diarrheic calves were measured, and diarrhea incidence from birth to weaning (1–60 days) was calculated based on fecal scores. Data revealed that the third week (W) was the peak period for the occurrence of pre-weaning calf diarrhea (Figure 2A), with 79.01% of cases occurring between weeks 1 to 4 (Figure 2B).

Growth performance indicators for calves aged 1–4 W, measured within 1 week post-diarrhea, included body weight (BW), average daily weight gain, withers height, chest circumference, hip cross height, hip height, hip width, body length, rump length, hucklebone width, and cannon bone circumference (Figures 2C–J). On day 14, D1W calves had significantly lower average daily weight gain and chest circumference compared to H1W calves (Figures 2C,G, *p* < 0.05). On day 21, BW, average daily weight gain, chest circumference, hip cross height, and body length of D2W calves were significantly lower than those of H2W calves (Figures 2D,H, *p* < 0.05). On day 28, D3W calves exhibited significantly lower average daily weight gain, body length, and cannon bone circumference compared to H3W calves (Figures 2E,I, *p* < 0.05). On day 36, BW, average daily weight gain, withers height, chest circumference, and hip height of D4W calves were significantly lower than those of H4W calves (Figures 2F,J, *p* < 0.05).

### 3.2 Analysis of the composition of the fecal microbiota of diarrheic and healthy calves

**Figure 3:**
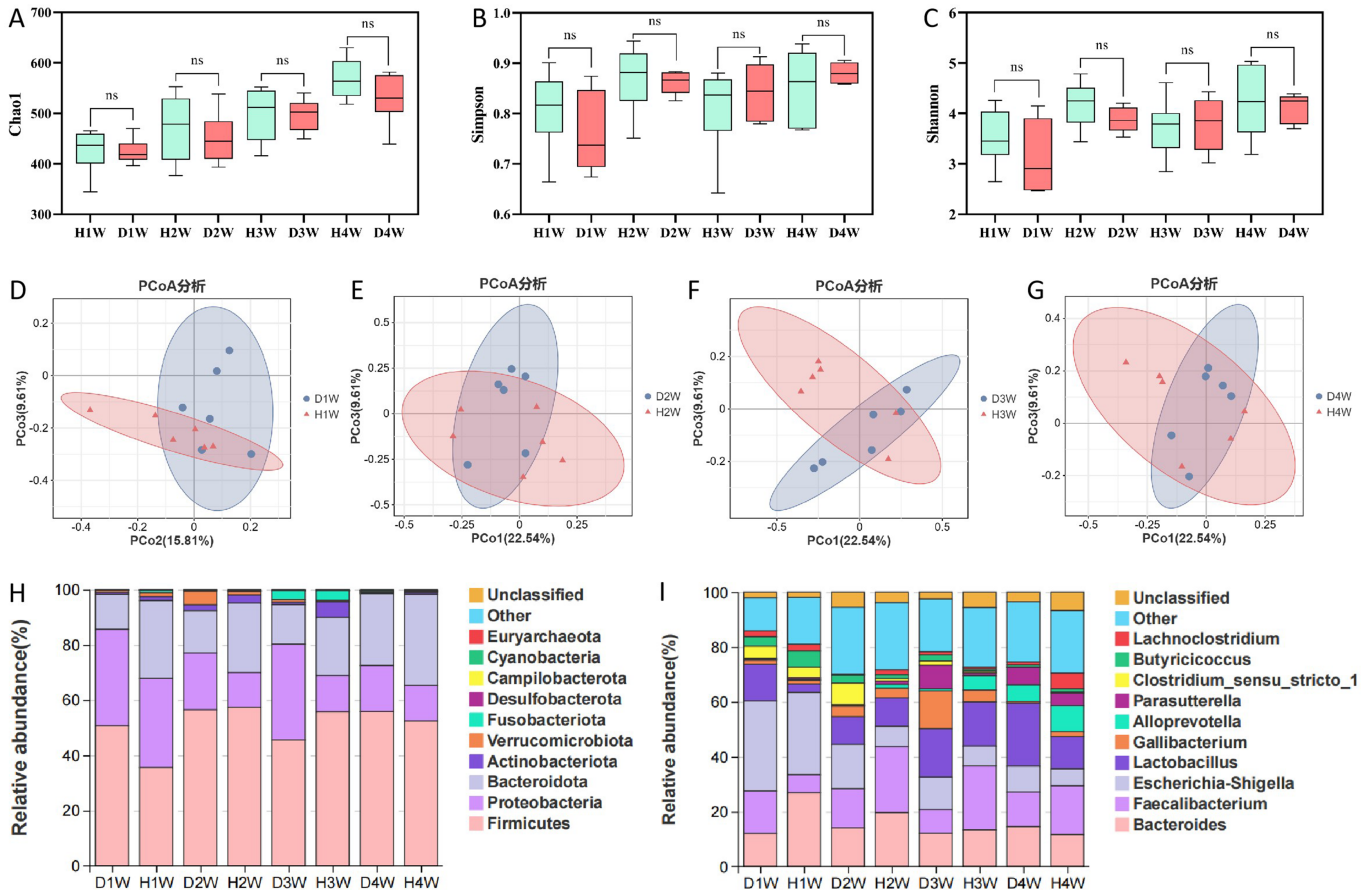
Differences in rectal microbiota between healthy and diarrheal calves. Chao 1 index **(A)**. Shannon index **(B)**. Simpsons index **(C)**. PCoA-analysis of D1W and H1W fecal flora of calves **(D)**. PCoA-analysis of D2W and H2W fecal flora of calves **(E)**. PCoA-analysis of D3W and H3W fecal flora of calves **(F)**. PCoA-analysis of D4W and H4W fecal flora of calves **(G)**. Histogram shows the top 10 relative abundances of the phylum in this taxa **(H)**. Histogram shows the top 10 relative abundances of the genus in this taxa **(I)**.

Fecal samples from diarrheic and healthy calves were analyzed using 16S rRNA sequencing. Chao1 (Figure 3A), Shannon (Figure 3B), and Simpson (Figure 3C) indices revealed lower microbial diversity in 1- to 4-week-old diarrheic calves compared to healthy calves. Principal coordinate analysis (PCoA) using the Bray-Curtis distance revealed significant differences in beta-diversity in fecal microbial communities between diarrheic and healthy calves (Figures 3D–G).

At the phylum level, Firmicutes, Proteobacteria, and Bacteroidota were predominant in both groups, with Firmicutes being the most abundant, accounting for 52.14 and 50.30% of the total microbial population in diarrheic and healthy calves, respectively (Figure 3H). Specifically, Firmicutes were more abundant in D1W calves than that in H1W calves, whereas Bacteroidota were less abundant. The abundance of Proteobacteria was significantly higher in the feces of D2W, D3W, and D4W calves compared to H2W, H3W, and H4W calves.

At the genus level, *Bacteroides*, *Faecalibacterium*, *Escherichia-shigella*, and *Lactobacillus* were the dominant genera in the fecal microbiota of both diarrheic and healthy calves (Figure 3I). *Escherichia-shigella* was the primary genus in D1W and D2W calves, accounting for 32.83 and 16.17% of total bacteria in the gut, respectively. It was also the main genus in H1W calves, comprising 30.5% of the total gut microbiota. Conversely, *Lactobacillus* was the most dominant genus in D3W and D4W calves, accounting for 17.68 and 22.80% of total bacteria in the gut, respectively (Figure 3I). *Faecalibacterium* was the predominant genus in H2W, H3W, and H4W calves, representing 24.08, 23.38, and 17.82% of the bacterial population in the gut, respectively (Figure 3I).

### 3.3 Analysis of differences in the fecal microbiota between healthy and diarrheic calves

**Figure 4:**
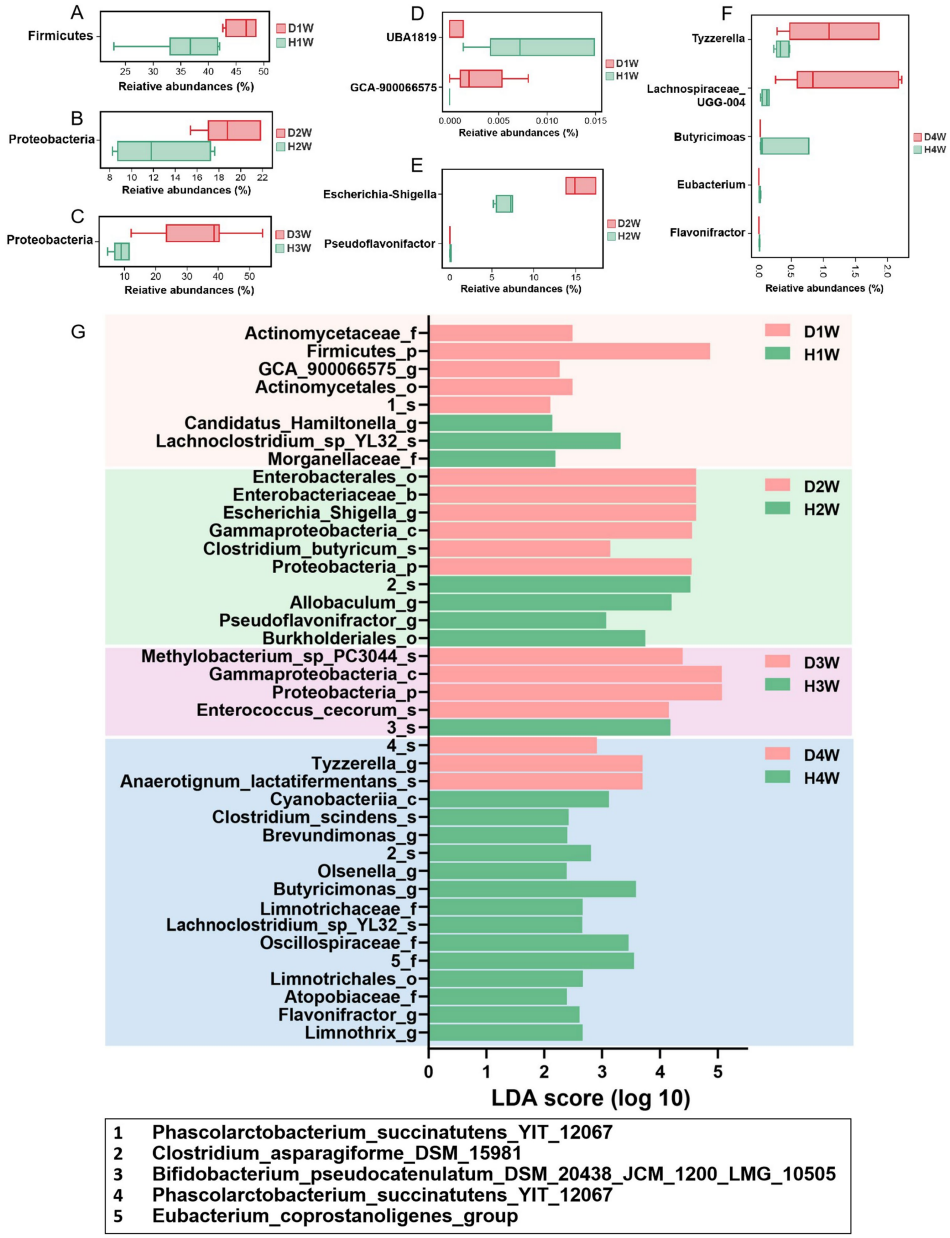
Significant differences in rectal flora between healthy and diarrheal calves. Wilcoxon rank sum test of D1W and H1W fecal flora of calves at Phylum level **(A)**. Wilcoxon rank sum test of D2W and H2W fecal flora of calves at the Phylum level **(B)**. Wilcoxon rank sum test of D4W and H4W fecal flora of calves at the Phylum level **(C)**. Wilcoxon rank sum test of D1W and H1W fecal flora of calves at genus level **(D)**. Wilcoxon rank sum test of D2W and H2W fecal flora of calves at genus level **(E)**. Wilcoxon rank sum test of D4W and H4W fecal flora of calves at genus level **(F)**.LEfSE analysis of fecal flora of D1W and H1W, D2W and H2W, D3W and H3W, D4W and H4W calves **(G)**.

The Wilcoxon rank sum test was used to analyze differences in the fecal microbiota of 1–4 W diarrheic and healthy calves. At the phylum level, Firmicutes were significantly increased in D1W compared to H1W calves (Figure 4A, *p* < 0.05), while Proteobacteria were significantly higher in D2W calves (Figure 4B, *p* < 0.05). Compared with H3W, Proteobacteria in the fecal microbiota of D3W calves were also significantly higher (Figure 4C, *p* < 0.05). No significant change was found in the fecal microbiota of D4W calves compared to H4W (*p* > 0.05).

At the genus level, *UBA1819* in the fecal microbiota of D1W calves was significantly decreased and *GCA-900066575* was significantly increased compared with H1W (Figure 4D, *p* < 0.05). Compared to H2W, *Escherichia-shigella* was found in significantly higher amounts in the fecal microbiota of D2W calves while *Pseudoflavonifractor* was found in significantly lower amounts (Figure 4E, *p* < 0.05). No significant changes were found in the fecal microbiota of D3W and H3W calves (*p* > 0.05). *Tyzzerella* and *Lachnospiraceae_UCG-004* in the fecal microflora of D4W calves were found significantly increased compared to H4W, while *Butyricimonas*, *Eubacterium*, and *Flavonifractor* were significantly decreased (Figure 4F, *p* < 0.05).

LEfSE analysis revealed that *Lachnoclostridium_sp_YL32* had the highest LDA score in the fecal microbiota of H1W calves (LDA = 3.68, Figure 4G, *p* < 0.05), whereas Firmicutes had the highest LDA score in the fecal microbiota of D1W calves (LDA = 4.87, Figure 4G, *p* < 0.05). In H2W calves, *Clostridium_asparagiforme_DSM_15981* had the highest LDA score (LDA = 4.52, Figure 4G, *p* < 0.05), whereas *Escherichia_shigella* had the highest LDA score in D2W calves (LDA = 4.62, Figure 4G, *p* < 0.05). *Bifidobacterium_pseudocatenulatum_DSM_20438_JCM_ 1200_LMG_10505* had the highest LDA score in H3W calves (LDA = 4.18, Figure 4G, *p* < 0.05), whereas Proteobacteria showed the highest LDA score in D3W calves (LDA = 5.07, Figure 4G, *p* < 0.05). *Butyricimonas* had the highest LDA score in H4W calves (LDA = 3.59, Figure 4G, *p* < 0.05), whereas *Tyzzerella* had the highest LDA score in D4W calves (LDA = 3.70, Figure 4G, *p* < 0.05).

Thus, *Escherichia_shigella*, *Tyzzerella*, and *Lachnospiraceae_UCG-004* and other differential microorganisms were significantly increased in diarrheic calves, suggesting that these microorganisms may be key contributors to promoting diarrhea. In contrast, *Eubacterium_coprostanoligenes_group*, *Pseudoflavonifractor*, *Butyricimonas*, and *Flavonifractor* were more prevalent in healthy calves and were found significantly reduced in diarrheic calves, indicating that these microorganisms may promote intestinal health and help prevent diarrhea in calves.

### 3.4 Functional analysis of the fecal microbiota in healthy and diarrheic calves

**Figure 5:**
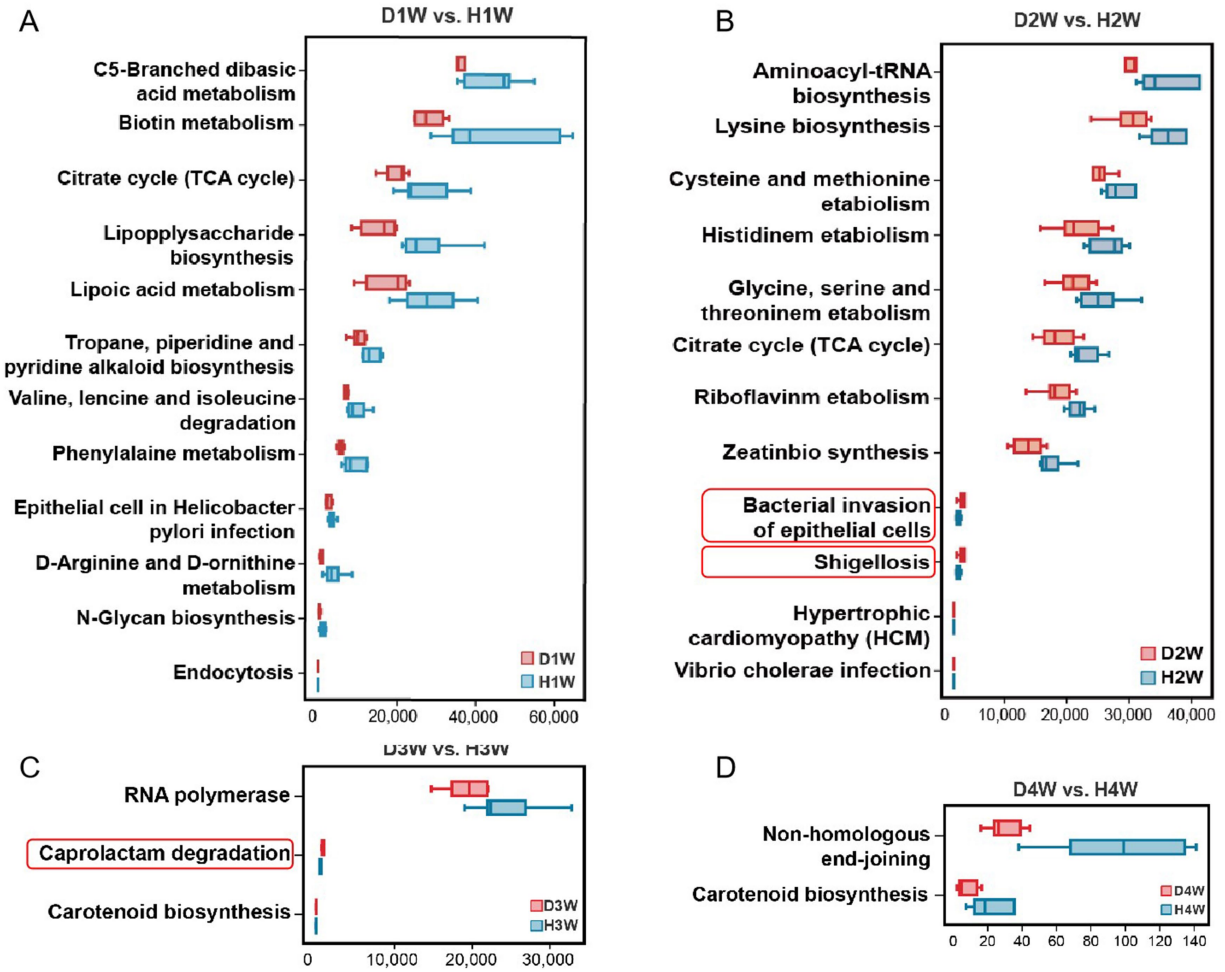
Functional analysis of intestinal microbiota difference between healthy and diarrheal calves. KEGG enrichment analysis of fecal metabolites in D1W and H1W calves **(A)**. KEGG enrichment analysis of fecal metabolites in D2W and H2W calves **(B)**. KEGG enrichment analysis of fecal metabolites in D3W and H3W calves **(C)**. KEGG enrichment analysis of fecal metabolites in D4W and H4W calves **(D)**.

KEGG pathway enrichment analysis revealed the enrichment of several pathways, including C5-branched dibasic acid metabolism, biotin metabolism, citrate cycle (TCA cycle), and lipopolysaccharide biosynthesis in both D1W and H1W calves (Figure 5A, *p* < 0.05). Moreover, pathways including aminoacyl-tRNA biosynthesis, lysine biosynthesis, cysteine and methionine metabolism, and histidine metabolism were significantly enriched in both D2W and H2W calves, but significantly reduced in D2W calves compared to H2W calves (Figure 5B, *p* < 0.05). Additionally, bacterial invasion of epithelial cells and shigellosis pathways were notably increased in D2W calves compared to H2W calves (Figure 5B, *p* < 0.05). In D3W and H3W calves, RNA polymerase, caprolactam degradation, and carotenoid biosynthesis were enriched, with RNA polymerase and carotenoid biosynthesis pathways significantly reduced in D3W calves compared to H3W calves, with caprolactam degradation increased (Figure 5C, *p* < 0.05). Moreover, non-homologous end-joining and carotenoid biosynthesis were enriched, but significantly reduced in D4W calves compared with H4W calves (Figure 5D, *p* < 0.05).

### 3.5 Correlation analysis of fecal microbial and growth performance in diarrheic calves

**Figure 6:**
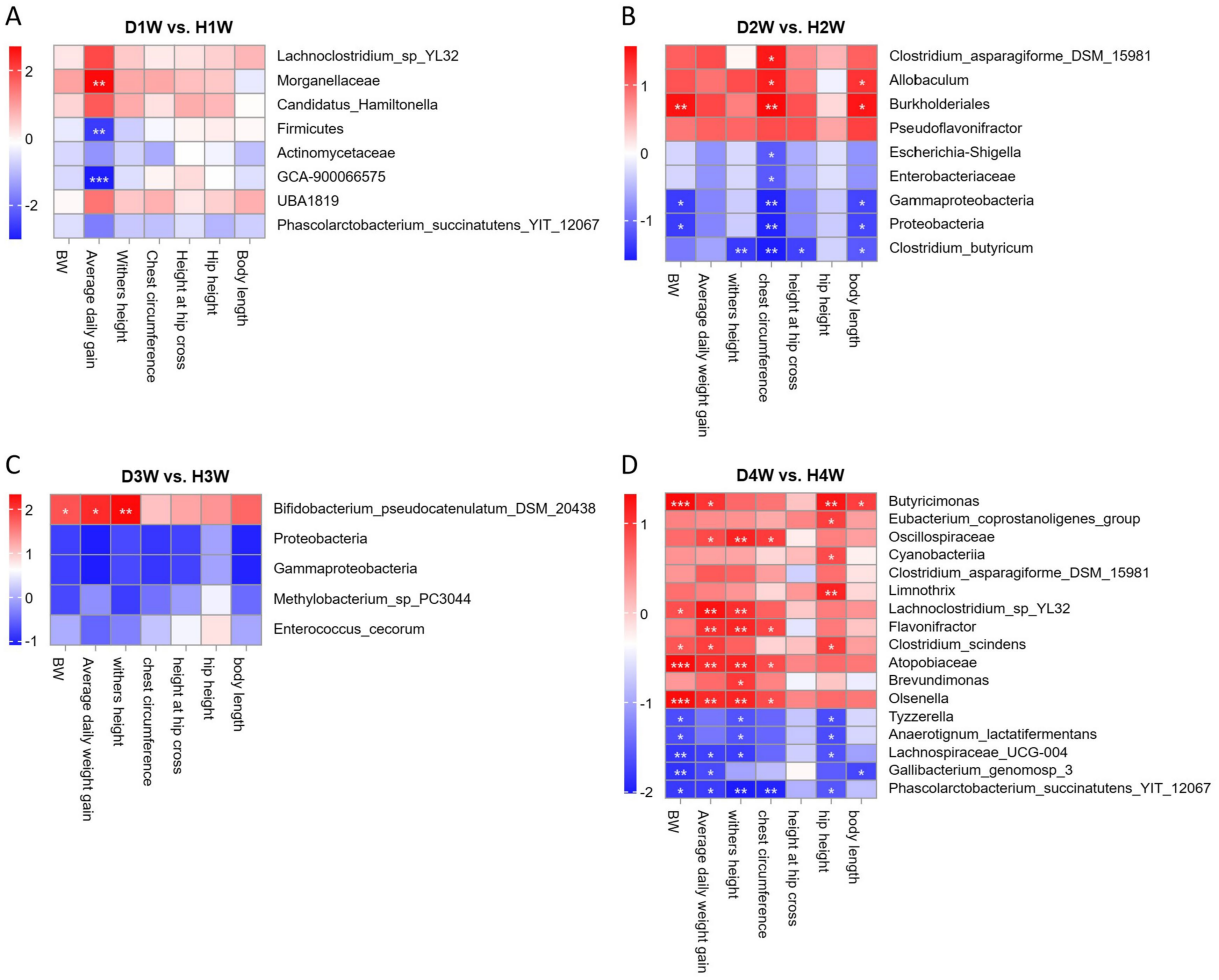
Relationship between fecal bacterial of calves with diarrhea and growth performance after 1 week of birth. Correlation of differential fecal bacterial in diarrheic calves at 1st week of age with 14 d growth performance of calves **(A)**. Correlation of differential fecal bacterial in diarrheic calves at 2 weeks of age with 21 d of calf growth **(B)**. Correlation of differential fecal bacterial in diarrheic calves at 3 weeks of age with calf growth at 28 d of age **(C)**. Correlation of differential fecal bacterial in diarrheic calves at 4 weeks of age with calf performance at 36 d **(D)**. *p < 0.05, **p < 0.01, ***p < 0.001.

Spearman correlation analysis was used to explore the relationship between differential fecal microbes and growth performance traits in calves at various time points during pre-weaning. The findings revealed that the average daily weight gain of D1W and H1W calves was significantly negatively associated with the relative abundances of *GCA-900066575* and Firmicutes, and significantly positively associated with *Morganellaceae* (Figure 6A, *p* < 0.05). For D2W and H2W calves, body weight and height were significantly negatively correlated with *Enterobacteriaceae*, *Escherichia_shigella*, *Gammaproteobacteria*, *Proteobacteria*, and *Clostridium_butyricum* (Figure 6B, *p* < 0.05), but significantly positively correlated with *Clostridium_asparagiforme_DSM_ 15981*, *Burkholderiales*, and *Allobaculum* (Figure 6B, *p* < 0.05). In D3W and H3W calves, BW, chest circumference, and withers height were significantly negatively correlated with *Proteobacteria*, *Gammaproteobacteria*, *Enterococcus_cecorum*, and *Methylobacterium_sp_PC3044*, but were significantly positively correlated with *Bifidobacterium_pseudocatenulatum_DSM_20438* (Figure 6C, *p* < 0.05). For D4W and H4W calves, growth performance was significantly negatively correlated with *Tyzzerella*, *Anaerotignum_lactatifermentans*, *Gallibacterium_genomosp_3*, *phascolarctobacterium_succinatutens_YIT_12067* and *Lachnospiraceae_UCG-004*, but significantly positively correlated with *Butyricimonas*, *Oscillospiraceae*, and *Lachnoclostridium_sp_YL32* (Figure 6D, *p* < 0.05). Moreover, the relative abundance of *GCA-900066575*, *Escherichia_shigella*, *Enterococcus_cecorum*, and *Tyzzerella* were significantly negatively correlated with calf growth performance, suggesting that these bacterial groups may inhibit growth in diarrheic calves.

### 3.6 Analysis of fecal metabolites in diarrheic calves

OPLS-DA was used to analyze fecal metabolites of 1–4 W diarrheic and healthy calves. The results of PCA plots (Supplementary Figures S2A–D) and OPLS-DA plots (Supplementary Figures S2I–L) indicated significant differences in fecal metabolites between the two groups. A Q2 regression line generated with alignment tests (Supplementary Figures S2I–L), demonstrating that the OPLS-DA model provided a good fit for distinguishing the fecal metabolite profiles of diarrheic and healthy calves.

### 3.7 Differential analysis of fecal metabolites in healthy and diarrheic calves

**Figure 7:**
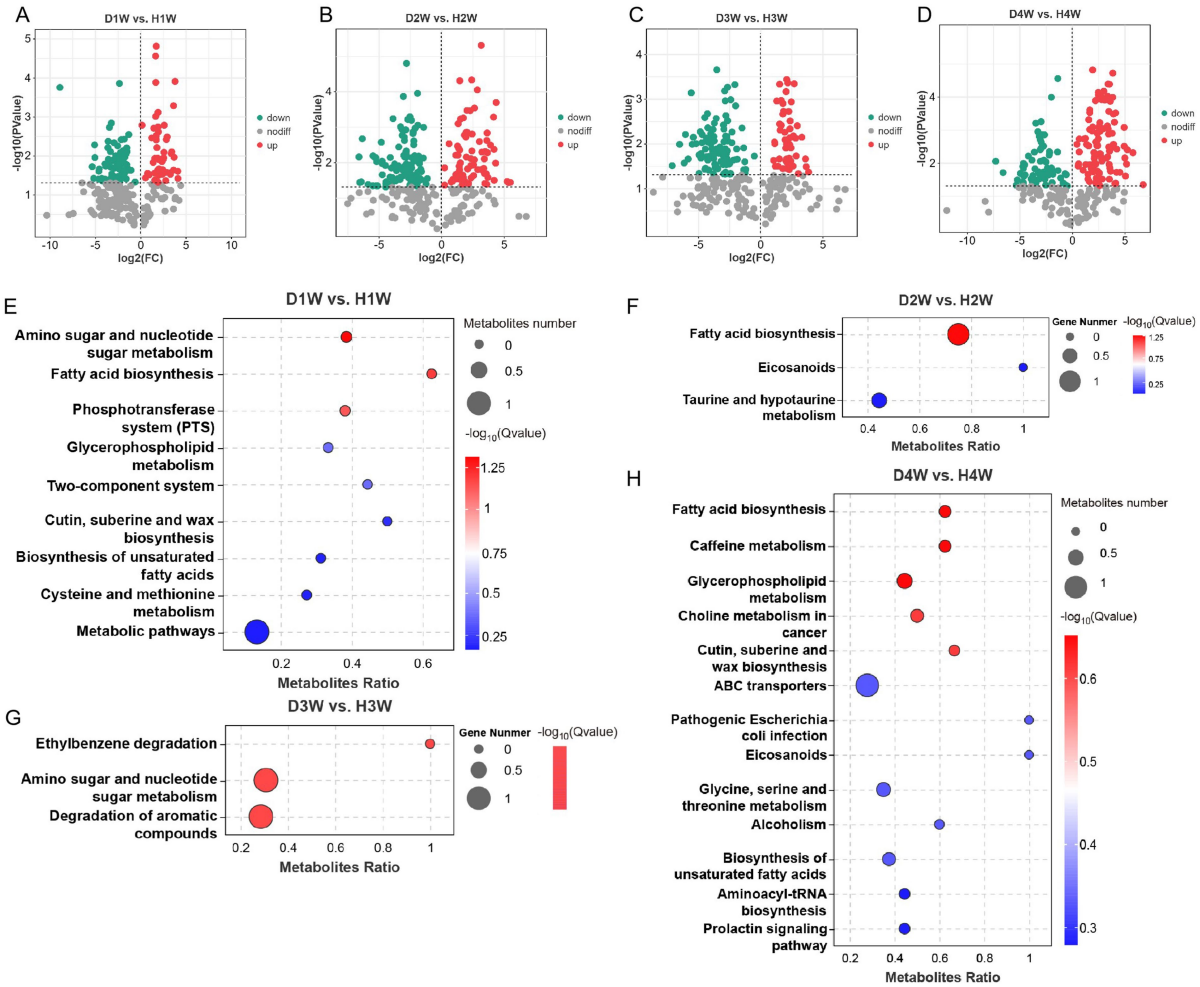
Differential expression of metabolites in feces between healthy and diarrheal calves. Volcano maps of differential metabolites in D1W and H1W calf feces **(A)**. Volcano maps of differential metabolites in D2W and H2W calf feces **(B)**. Volcano maps of differential metabolites in D3W and H3W calf feces **(C)**. Volcano maps of metabolite differences in D4W and H4W calf feces **(D)**. Bubble diagram of KEGG metabolic pathways with significant enrichment of differential metabolites in D1W and H1W calf feces **(E)**. Bubble diagram of KEGG metabolic pathways with significant enrichment of differential metabolites in D2W and H2W calf feces **(F)**. Bubble diagram of KEGG metabolic pathways with significant enrichment of differential metabolites in D3W and H3W calf feces **(G)**. Bubble diagram of KEGG metabolic pathways with significant enrichment of differential metabolites in D4W and H4W calf feces **(H)**.

A differential analysis of fecal metabolites between 1 and 4 W diarrheic and healthy calves revealed 136 differential metabolites in D1W versus H1W calves, of which 49 were up-regulated and 87 were down-regulated (Figure 7A; Supplementary Table S5, VIP > 2, *p* < 0.05). For D2W versus H2W, 182 metabolites were differentially expressed, of which 76 were up-regulated and 106 down-regulated (Figure 7B; Supplementary Table S6, VIP > 2, *p* < 0.05). A total of 156 differential metabolites were found between D3W and H3W calves, of which 54 up-regulated and 102 down-regulated (Figure 7C; Supplementary Table S7, VIP > 2, *p* < 0.05). Finally, for D4W and H4W calves, 172 differential metabolites were found (Figure 7D; Supplementary Table S8, VIP > 2, *p* < 0.05), of which 102 up-regulated and 70 down-regulated.

KEGG enrichment analysis was performed on differential metabolites for selected calf fecal samples. It was found that metabolites from D1W and H1W fecal samples were significantly enriched in pathways related to amino sugar and nucleotide sugar metabolism, fatty acid biosynthesis, phosphotransferase system (PTS), glycerophospholipid metabolism, two-component system, cutin, suberine and wax biosynthesis, biosynthesis of unsaturated fatty acids, cysteine and methionine metabolism, and other metabolic pathways (Figure 7E, *p* < 0.05). Metabolites from D2W and H2W calves were significantly enriched in pathways related to fatty acid biosynthesis, eicosanoids, and taurine and hypotaurine metabolism (Figure 7F, *p* < 0.05). Differential metabolites in D3W and H3W calves were significantly enriched in pathways related to ethylbenzene degradation, amino sugar and nucleotide sugar metabolism, degradation of aromatic compounds (Figure 7G, *p* < 0.05). Moreover, differential metabolites in D4W and H4W calves were significantly enriched in pathways related to fatty acid biosynthesis, caffeine metabolism, glycerophospholipid metabolism, choline metabolism in cancer, cutin, suberine and wax biosynthesis, ABC transporters, pathogenic *E. coli* infection, eicosanoids, glycine, serine and threonine metabolism, alcoholism, biosynthesis of unsaturated fatty acids, aminoacyl-tRNA biosynthesis, and prolactin signaling pathway (Figure 7H, *p* < 0.05).

In total, 136, 182, 156, and 172 fecal metabolites were identified between D1W versus H1W, D2W versus H2W, D3W versus H3W, and D4W versus H4W, respectively, which could be considered key metabolites contributing to the onset of diarrhea in calves, and may serve as biomarkers for the condition.

### 3.8 Correlation analysis of fecal metabolites and growth performance in diarrheic calves

**Figure 8:**
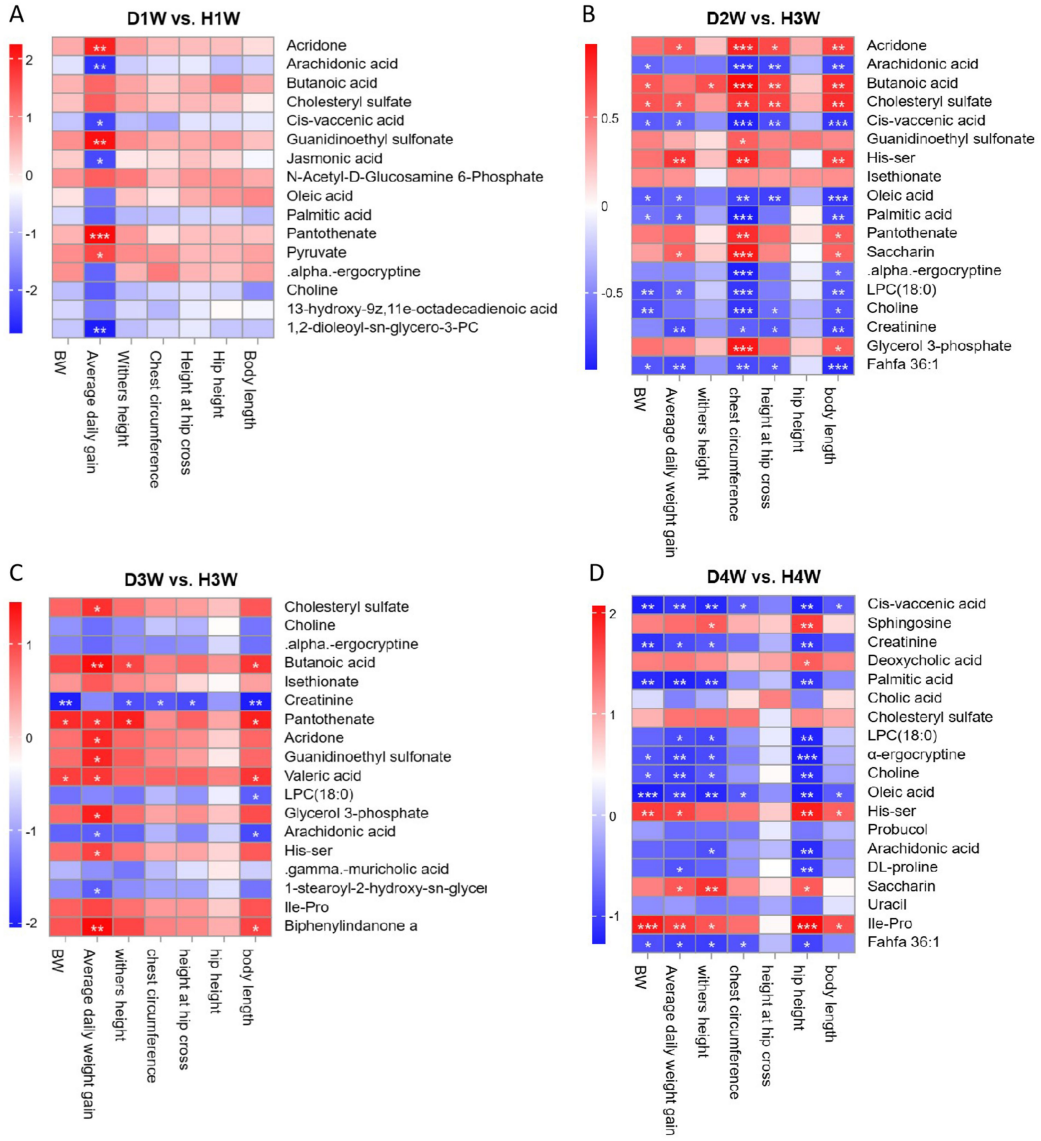
Relationship between fecal metabolites and growth performance of calves with diarrhea. Correlation between differential fecal metabolites of D1W and H1W calves and 14d growth performance of calves **(A)**. Correlation between D2W and H2W calf fecal differential metabolites and 21-day growth performance of calves **(B)**. Correlation between D3W and H3W calf fecal differential metabolites and 28-day growth performance of calves **(C)**. Differential fecal metabolites of D4W and H4W calves were correlated with 36-day growth performance of calves **(D)**. *p < 0.05, **p < 0.01, ***p < 0.001.

Spearman correlation analysis revealed significant relationships between fecal differential metabolites and growth performance traits (e.g., BW, average daily gain, body height, chest circumference, cross section height, and body slant length) in 1–4 W diarrheic calves (Figure 8). The results showed that the average daily gain of diarrheic calves on the first week of birth was significantly positively correlated with acridone, pantothenate, guanidinoethyl sulfonate, and pyruvate (Figure 8A, *p* < 0.05), but significantly negatively correlated with arachidonic acid, 1, 2-dioleoyl-SN-glycero-3-PC, and cis-vaccenic acid (Figure 8A, *p* < 0.05). On the second week, chest circumference and body length of diarrheic calves were significantly negatively correlated with arachidonic acid, oleic acid, pantothenate and cis-vaccenic acid, choline, creatinine, palmitic acid, His-Ser, glycerol 3-phosphate, *α*-ergocryptine and 1-stearoyl-sn-glycerol 3-phosphocholine [LPC(18:0)] (Figure 8B, *p* < 0.05), but significantly positively correlated with butanoic acid, acridone, cholesteryl sulfate, His-Ser, saccharin, pantothenate, and glycerol 3-phosphate (Figure 8B, *p* < 0.05). Growth performance of diarrheic calves on the third week of birth was significantly negatively correlated with choline, LPC (18:0), arachidonic acid, 1-stearoyl-2-hydroxy-sn-glycero-3-PE, gamma-muricholic acid, α-ergocryptine, and creatinine (Figure 8C), while average daily weight gain and body length were significantly positively correlated with butanoic acid, biphenylindanone A, pantothenate, and valeric acid (Figure 8C, *p* < 0.05). Finally, on the fourth week of birth, average daily weight gain, withers height, and hip height of diarrheic calves were significantly negatively correlated with Cis-vaccenic acid, oleic acid, choline, fahfa 36:1, palmitic acid, LPC (18:0), α-ergocryptine and creatinine (Figure 8D, *p* < 0.05), while growth performance was significantly positively correlated with sphingosine, saccharin, cholesteryl sulfate, deoxycholic acid, His-Ser and Ile-Pro (Figure 8D).

Thus, it was found that the above fecal metabolites were positively correlated with growth performance traits in calves during the first 28 days birth. In contrast, metabolites such as arachidonic acid, Cis-vaccenic acid, choline, creatinine, α-ergocryptine, and LPC (18:0) were negatively correlated with growth performance in calves. Hence, these metabolites may be considered key factors promoting diarrhea and hindering growth in calves during the first 28 days post-birth.

### 3.9 Weighted gene co-expression network analysis (WGCNA) of fecal metabolites in diarrheic calves

WGCNA was performed on fecal metabolites of 1–4 W diarrheic and healthy calves, and four groups of data were created considering diarrheic and healthy experimental groups, with diarrhea (D) and health (H) as traits.

**Figure 9:**
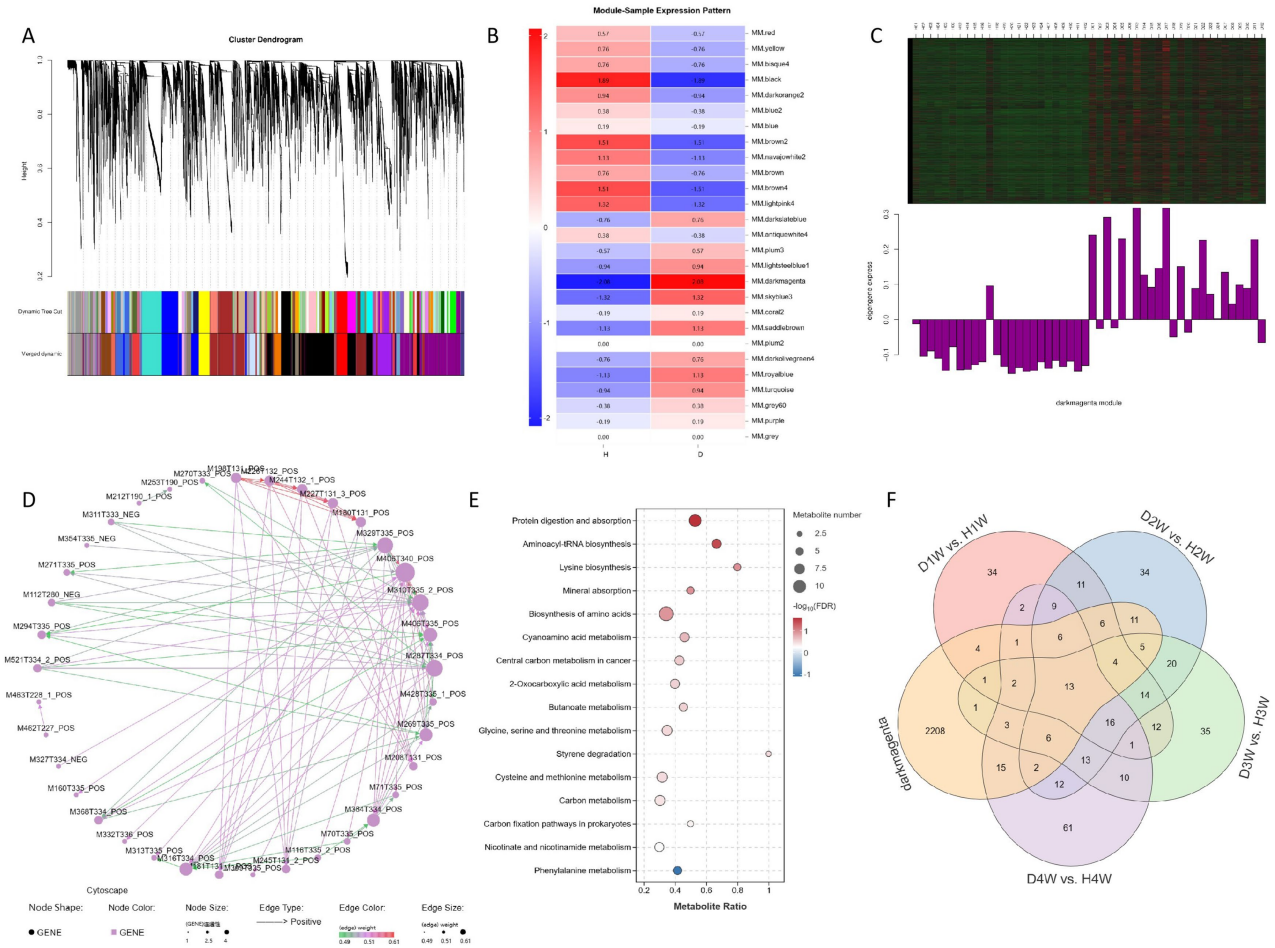
WGCNA analysed the changes of fecal metabolites of diarrheal and healthy cows. Weighted co-expression network **(A)**. Metabolite module association heat map **(B)**. Correlation heat map of character modules **(C)**. Metabolite regulatory network map **(D)**. KEGG enrichment analysis **(E)**. Venn diagrams **(F)**.

A total of 15,728 metabolites were identified in the original data. Using a selected power of 8, a weighted co-expression network model was constructed. These 15,728 metabolites were categorized into 20 modules, with the gray module consisting of metabolites that could not be assigned to any specific group (Figure 9A). Spearman correlation algorithm was used to calculate the correlation coefficients and *p*-values between characteristic metabolites and disease traits, generating a correlation heat map. The results indicated that the darkmagenta module was significantly positively correlated with disease traits (Figure 9B, *p* < 0.05). Cluster analysis of the metabolite expression within the module revealed substantial differences in fecal metabolite expression between diarrheic and healthy calves (Figure 9C). Furthermore, the regulatory network diagram of the darkmagenta module revealed fecal metabolites such as fluazifop-butyl, dehydrocurvularin, and 4′,5-dihydroxy-7-methoxyflavanone as hub components (Figure 9D). KEGG enrichment analysis of fecal metabolites from this module revealed significant enrichment in pathways related to protein digestion and absorption, aminoacyl-tRNA biosynthesis, and lysine biosynthesis (Figure 9E).

Furthermore, a Venn diagram comparing differential fecal metabolites in the darkmagenta module and 1–4 W diarrheic calves was drawn. In total, 37 fecal metabolites were commonly found in 1 W diarrheic and healthy calves; 53 in 2 W calves; 35 in 3 W calves; and 48 in 4 W calves. Considering these, it was found that 13 differential fecal metabolites were consistently shared across all group comparisons (Figure 9F; Table 1).

**Table 1:** Common metabolites of the darkmagenta module with diarrhoea and healthy calves at 1–4 weeks of age.

ID	MS2_name
M303T50_2_NEG	Arachidonic acid (peroxide free)
M496T221_4_POS	.alpha.-ergocryptine
M787T170_POS	1,2-dioleoyl-sn-glycero-3-phosphatidylcholine
M480T222_3_NEG	1-stearoyl-2-hydroxy-sn-glycero-3-phosphoethanolamine
M759T172_1_POS	1-palmitoyl-2-linoleoyl-sn-glycero-3-phosphocholine
M171T54_NEG	Capric acid
M811T165_POS	1-stearoyl-2-arachidonyl-sn-glycero-3-phosphocholine
M329T49_NEG	(z)-9,12,13-trihydroxyoctadec-15-enoic acid
M733T174_1_POS	1-oleoyl-2-myristoyl-sn-glycero-3-phosphocholine
M199T52_NEG	Dodecanoic acid
M992T221_POS	1-palmitoyl-sn-glycero-3-phosphocholine
M722T52_NEG	Pg 32:0
M845T164_NEG	Pc(17:1/9-hode)

Subsequently, KEGG enrichment analysis of these 13 fecal metabolites revealed that, compared with the whole metabolome, these metabolites were mainly significantly enriched in fatty acid biosynthesis, linoleic acid metabolism, amoebiasis, and GnRH signaling pathway. These findings suggest that these 13 differential fecal metabolites may play a key role in promoting diarrhea in calves.

### 3.10 Correlation analysis of fecal microorganisms and metabolites in diarrheic calves

**Figure 10:**
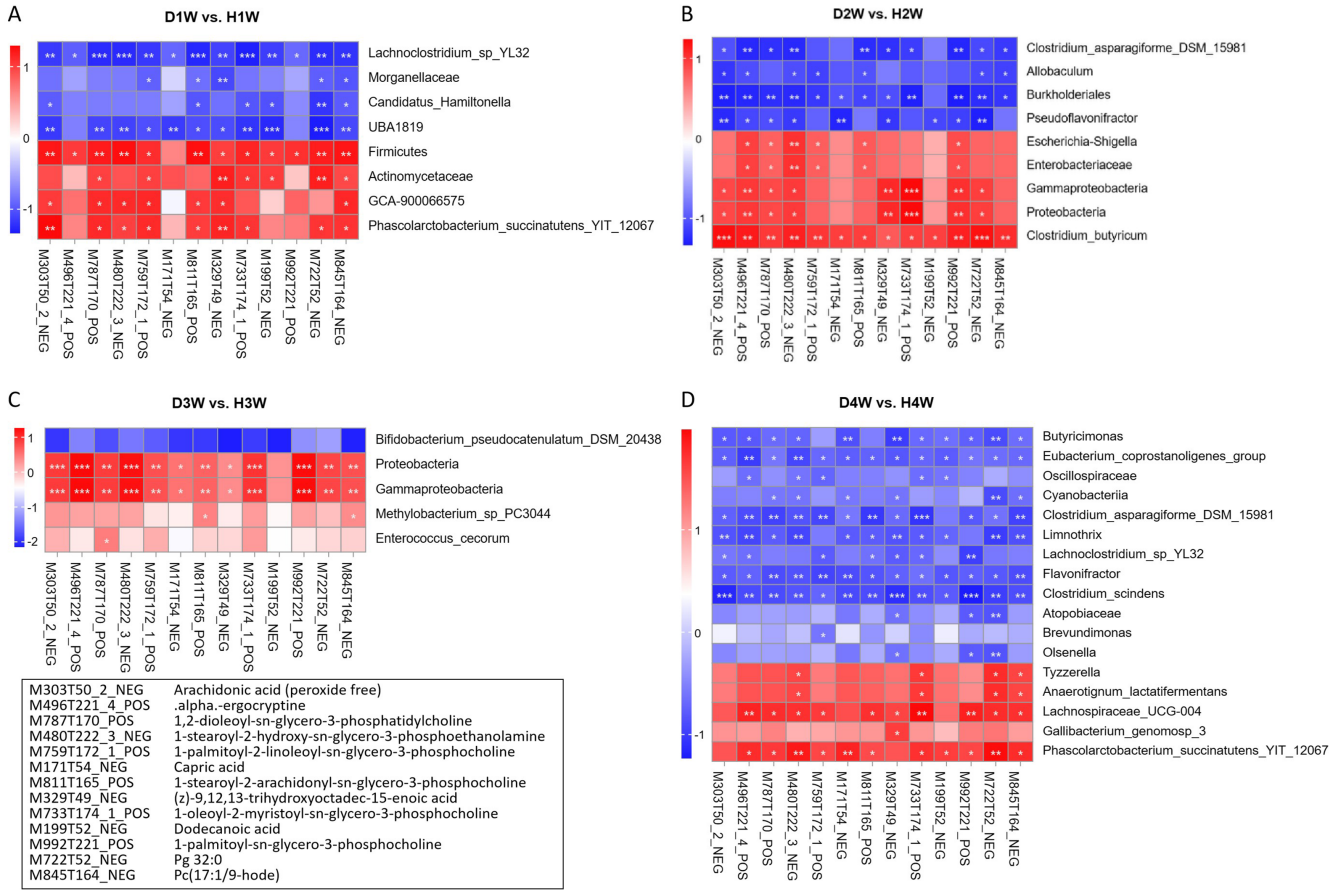
Correlation analysis of intestinal microorganisms and fecal metabolites in calves with diarrhea. Correlation between differential fecal microbes and differential metabolites in D1W and H1W calves **(A)**. Correlation between different fecal microorganisms and different metabolites in D2W and H2W calves **(B)**. Correlation between differential fecal microbes and differential metabolites in D3W and H3W calves **(C)**. Correlation between different fecal microbes and different metabolites in D4W and H4W calves **(D)**. *p < 0.05, **p < 0.01, ***p < 0.001.

Spearman correlation analysis was performed to explore the relationship between fecal microorganisms in diarrheic calves and the 13 common differential metabolites identified using WGCNA. The results showed that Firmicutes, Actinomycetaceae, *GCA-900066575* and *Phascolarctobacterium_succinatutens_YIT_12067* in D1W vs. HIW calves were positively correlated with the identified 13 differential fecal metabolites, while *Lachnoclostridium_sp_YL32*, *Morganellaceae*, *Candidatus_hamiltonella* and *UBA1819* were negatively correlated (Figure 10A). *Clostridium_asparagiforme_DSM_15981*, *Allobaculum*, *Burkholderiales* and *Pseudoflavonifractor* in D2W vs. H2W calves were negatively correlated with the 13 fecal metabolites, while *Escherichia-shigella*, *Enterobacteriaceae*, Gammaproteobacteria, Proteobacteria and *Clostridium_butyricum* were positively correlated (Figure 10B). *Bifidobacterium_pseudocatenulatum_DSM_20438* in D3W vs. H3W calves was negatively correlated with these 13 fecal metabolites, while Proteobacteria, Gammaproteobacteria, *Methylobacterium_sp_PC3044* and *Enterococcus_cecorum* were positively correlated (Figure 10C). *Butyricimonas*, *Oscillospiraceae*, and *Eubacterium_coprostanoligenes_group* in D4W vs. H4W calves were negatively correlated with these 13 fecal metabolites, while *Tyzzerella*, *Lachnospiraceae_UCG-004*, *Anaerotignum_lactatifermentans*, *Phascolarctobacterium_succinatutens_YIT_12067* and *Gallibacterium_genomosp_3* were positively correlated (Figure 10D).

Hence, *GCA-900066575*, *Escherichia-shigella*, *Enterococcus_cecorum*, *Methylobacterium_sp_PC3044*, *Lachnospiraceae_UCG-004*, and *Tyzzerella* in calf feces within 1–4 W of birth were positively correlated with these 13 fecal metabolites, suggesting that these microorganisms may be key in promoting diarrhea in calves during this critical period.

## 4 Discussion

### 4.1 Changes in fecal microbiota of calves

Diarrhea is a critical factor contributing to the disruption of the intestinal microbiota in calves (He et al., 2022). Previous studies have shown that diarrhea induces dysbiosis in the intestinal microbiota of calves, altering its structure and composition compared to healthy calves (Gomez et al., 2017). Once the intestinal barrier is damaged, pathogenic bacteria can colonize the intestinal tract, triggering inflammation (Qin et al., 2010). Diarrheic calves often exhibit a higher abundance of opportunistic pathogens, particularly members of Proteobacteria and Fusobacteriota, which are frequently associated with the dysregulation of the intestinal microbiota (Fan et al., 2021; Zeineldin et al., 2018). It has been shown that the main bacteria associated with high incidence of diarrhea belong the phyla Firmicutes (78.93%), Fusobacteriota (7.75%), Actinobacteriota (5.99%), and Proteobacteria (5.15%), with Firmicutes and Bacteroidetes being dominant in pre-weaning diarrhea calves, accounting for 80% (Kim E. T. et al., 2021). In the present study, it was found that Firmicutes (52.14%), Proteobacteria (26.74%), and Bacteroidota (17.09%) were the most prevalent bacteria in calves with diarrhea, which was consistent with the findings of previous studies. In particular, Firmicutes were the predominant phylum in 1–4 W diarrheic calves accounting for 52.14 and 50.30% of total bacteria in diarrheic calves and healthy calves, respectively.

As calves mature, on the day before weaning, Bacteroidota levels increase in the colon and cecum (Dias et al., 2018). It has been shown that *Alloprevotella* can be used as a probiotic to reduce or prevent diarrhea in calves (Rey et al., 2014). In the present study, *Alloprevotella* first appeared in H2W calves and its levels gradually increased, suggesting that this bacterial species can decelerate the occurrence of diarrhea in calves. *Enterobacteriaceae* was the dominant bacterial family in all fecal samples of calves (Klein-Jobstl et al., 2019). In particular, *Enterobacteriaceae* was found at 3–5 weeks of age in calves, but disappeared after the fifth week and was no longer detected in the subsequent weeks (Uyeno et al., 2010). Notably, in the present study, *Enterobacteriaceae* was detected in 2 W calves and was significantly increased in D2W diarrheic calves.

### 4.2 Intestinal microbiota and calf diarrhea

Changes in microbial dynamics can predict early-life diarrhea and enhance calf health (Bartels et al., 2010; Kim H. S. et al., 2021). Therefore, instability and low microbial diversity serve as potential biomarkers for predicting diarrhea in calves. Nutritional metabolic disorders, often linked to microecological disturbances, are marked by significant downregulation in gene expression related to ribosome translation, amino acid metabolism, and carbohydrate metabolism (Zeineldin et al., 2018). In particular, changes in amino acid metabolism have been identified as an important factor associated with calf diarrhea (Kim E. T. et al., 2021).

Energy metabolism is essential for body growth and development. When the energy levels cannot meet bodily needs, feed conversion and growth performance will be negatively impacted. Energy metabolism has been found to be positively correlated with the relative abundance of *Prevotella*, *Bacteroides*, and *Sutterella* in the gut (Lu et al., 2022). *Prevotella* can degrade starch, monosaccharides, and other non-cellulosic polysaccharides, produce volatile fatty acids to provide energy for the body and promote rumen development (Purushe et al., 2010). Similarly, *Bacteroides* can synthesize SCFAs. However, when the abundance of Bacteroidota in the gut is low, animals begin to produce *δ*-aminolevulinic acid with the support of the TCA cycle using glycine and succinyl coenzyme A as substrates. Thus, δ-aminolevulinic acid can be used as a marker to identify an increase in the abundance of *Proteobacteria*, which can lead to microecological dysregulation (Gomez et al., 2017).

Firmicutes are considered one of the primary producers of SCFAs, being more effective than Bacteroidota in promoting nutrient absorption (Li et al., 2021). Both Firmicutes and Bacteroidota participate in the degradation of complex polysaccharides, which allows the metabolization of carbohydrates, sugars, and fatty acids. However, a reduction in their abundance can impair these functions (Jami et al., 2013). Moreover, a significant negative correlation has been described between the abundance of *Lachnospiraceae* and the duration of diarrhea in calves. *Lachnospiraceae*, belonging to the phylum Firmicutes, can produce large amounts of SCFAs (Chen et al., 2017; Reichardt et al., 2014) and other metabolites (such as tryptophan), which help regulate immune and inflammatory responses (Arpaia et al., 2013; Smith et al., 2013). These metabolites decrease the levels of pro-inflammatory cytokines, such as tumor necrosis factor-*α*, interleukin-6, and interferon-*γ*, while increasing the production of anti-inflammatory factors in calves.

The Firmicutes/Bacteroidetes ratio is crucial for maintaining intestinal homeostasis in calves, and an imbalance in this ratio is linked to the onset of certain diseases (Cui et al., 2013; Stojanov et al., 2020). In particular, a significant increase in the Firmicutes/Bacteroidetes ratio leads to disturbances in the intestinal microbiota (Wang J. et al., 2019). In the present study, Firmicutes were found significantly increased in D1W diarrheic calves. At the species level, *Enterococcus_cecorum* within the phylum Firmicutes was found significantly increased in D3W diarrheic calves. At the genus level, *Lachnospiraceae_UCG-004* and *Tyzzerella*, and *Anaerotignum_lactatifermentans* at the species level, all belonging to the phylum Firmicutes, were found significantly increased in D4W diarrheic calves. Thus, the increase in the above taxa may be the primary contributors to diarrhea in D4W calves in this specific farm. Furthermore, the above findings revealed that the abundance of Firmicutes in the feces of diarrheic calves may be the primary cause of disturbances in the intestinal microbiota of calves, and could be considered the main cause of diarrhea of calves.

Conversely, *Clostridium_sp_YL32* belonging to the family *Lachnospiraceae* was found significantly decreased in D1W and D4W diarrheic calves, while *Clostridium_scindens* was significantly decreased in D4W diarrhea calves. Thus, these taxa can be considered beneficial bacteria that might contribute to hinder the onset of diarrhea in calves. Based on these observations, we speculated that the rapid immune response in calves during the initial stages of diarrhea may involve regulating inflammation through the modulation of *Lachnospiraceae* levels in the gut.

*Ruminococcaceae*, another family of butyrate-producing bacteria, can be used to predict and potentially prevent colic, with the Firmicutes (particularly *Lachnospiraceae* and *Ruminococcaceae*)/Proteobacteria ratio serving as a potential indicator (Weese et al., 2015). Although certain members of the family *Ruminococcaceae* are associated with diarrhea, others, such as *UBA1819*, a beneficial species belonging to the phylum Firmicutes, family *Rumenococcaceae*, have been linked to calf health, highlighting its potential to be used as probiotic strains in this particular commercial farm with this specific microbial colonization pattern (Chen et al., 2022). In the present study, compared with D2W, *UBA1819* was significantly increased in H1W calves, which indicated that *UBA1819* could be used as beneficial bacteria to promote calf growth. However, *GCA_900066575*, belonging to the phylum Firmicutes, was significantly increased in D1W diarrheic calves. Taken together, these findings highlight that Firmicutes played a pivotal role in disrupting the intestinal microbiota in calves.

In this study, *Escherichia-shigella* and *Gammaproteobacteria* were found to the main causes of diarrhea in D2W calves. *Escherichia-shigella* has been described to be associated with diarrhea-related diseases in calves in various studies (Akashi et al., 2017; Qu et al., 2012). *Escherichia-shigella* alter the intestinal environment of calves, promoting anaerobic microbial colonization and reducing the abundance of beneficial bacteria (Mayer et al., 2012). Intestinal colonization by *Escherichia-shigella* in calves likely increases the susceptibility of calves to *E. coli-*induced diarrhea, which is a well-known pathogen responsible for diarrhea in calves (Huang et al., 2020).

Proteobacteria, known for the production of lipopolysaccharide (LPS) that trigger inflammation, was the most abundant bacterial phylum in the intestinal microbiota of calves suffering from moist heat diarrhea (Mukhopadhya et al., 2012). However, the pathogenesis of Proteobacteria in the aggravation of diarrhea in calves is still unclear. In the present study, Proteobacteria was found to be the main phylum causing diarrhea in D2W calves, being significantly increased in D2W and D3W diarrheic calves, with *Escherichia-shigella* and *Gammaproteobacteria* identified as major contributors. Proteobacteria was the main bacterial group responsible for inflammation in D2W and D3W diarrheic calves, especially *Enterococcus_cecorum*, *Gammaproteobacteria*, and *Methylobacterium_sp_PC3044*.

*Bifidobacterium* detected in healthy calves is an important core and beneficial physiological flora in the intestinal tract of humans and animals, which has a significant impact on the early development of the body. Notably, *Bifidobacterium_pseudocatenulatum_DSM_20438_=_JCM_1200_=_LMG_10505* was significantly decreased in D3W diarrheic calves, which reinforces its role as a beneficial bacterium hindering the onset of diarrhea and promoting growth in calves.

### 4.3 Fecal metabolites and calf diarrhea

Polysaccharides, a key carbon source for intestinal microorganisms, are fermented into SCFAs including acetic acid, propionic acid, butanoic acid, valeric acid, and hexanoic acid, which play a critical role in maintaining mucosal integrity, metabolism, and immunity *in vivo*, thus promoting the growth of beneficial microorganisms in the gut. A reduction in SCFA-producing microbial species in the gut leads to compromised immune function (Angelini et al., 2014). For instance, propionic acid exerts immunomodulatory properties, inducing the expression of regulatory T cells (Duscha et al., 2020; Wang J. J. et al., 2019). The decrease in certain SCFA-producing bacteria, such as *Parabacteroides* and *Ruminococcus*, has been closely linked to reduced levels of acetic acid, whereas a decrease in *Parabacteroides*, *Ruminococcus*, *Fournierella*, and *Rikenellaceae_RC9_gut_group* has been shown to be closely related to a decrease in isocaproic acid content (Cui et al., 2023).

Our findings revealed that the contents of propionic acid, butanoic acid (D1W, D2W and D3W), and valeric acid (D3W group) were significantly lower in the feces of 1–4 W diarrheic calves compared to those in healthy calves. In addition, propionic acid and isobutyric acid were found to be significantly positively correlated with *Collinsella* and *Bifidobacterium*. It has been shown that changes in gut microbiota composition, especially related to a decrease in butyrate-producing bacteria, can lead to diarrhea in calves during the first week of birth (Gomez et al., 2017; Oikonomou et al., 2013). Acetate produced by Bacteroidota can be utilized by other bacteria (e.g., *Bactericoccus* and *Megamonas*) to produce butyrate and propionate (Trachsel et al., 2018), both of which are main sources of energy for intestinal epithelial cells in calves. Butyrate can also inhibit the signaling pathway of pro-inflammatory factors and enhance intestinal barrier function by increasing mucin secretion and strengthening tight junctions (Jonkers et al., 2012; Morgan et al., 2012).

*Butyricimonas* is a beneficial SCFA-producing bacterium that helps reduce inflammation. Our findings revealed that the concentration of *Butyricimonas* was significantly reduced in diarrheic calves with 4 weeks of age, while butanoic acid was significantly decreased in D1W, D2W, and D3W diarrheic calves. Moreover, butanoic acid was significantly negatively correlated with Proteobacteria in D3W diarrheic calves, and with Gammaproteobacteria. In addition, butanoic acid was significantly negatively correlated with Proteobacteria in D3W diarrheic calves and with Gammaproteobacteria in Proteobacteria; butanoic acid was negatively correlated with *Escherichia-shigella* within the class Gammaproteobacteria in D3W calves, as well as with growth performance traits in calves (e.g., BW, chest circumference, etc.).

Glycerophospholipids were a major component of the cell membrane and can help alleviate diarrhea-related diseases in calves (Akashi et al., 2017; Kalenyak et al., 2019). Phosphocholines (PCs) are involved in glycerophospholipid metabolism. In this experiment, 13 common differential metabolites associated with calf diarrhea traits were identified by WGCNA. The contents of glycerophospholipids (e.g., 1-palmitoyl-2-linoleoyl-sn-glycero-3-PC, 1-stearoyl-2-arachidonyl-sn-glycero-3-PC, 1-oleoyl-2-myristoyl-sn-glycero-3-phosphocholine, 1-palmitoyl-sn-glycero-3-PC, and 1-stearoyl-2-hydroxy-sn-glycero-3-phosphoethanolamine) were significantly elevated in diarrheic calves across all weeks, and were enriched in glycerol phospholipid metabolic pathway. The elevated levels of PC indicate that oxidative stress occurs in calves (Amin et al., 2023), and damaged intestinal mucosa triggers the release of arachidonic acid, which further promotes the release of reactive of oxygen species (ROS) and aggravates oxidative stress (Sanchez, 2018). In particular, lysophosphatidyl ethanolamine (LysoPE) and lysophosphatidylcholine (LysoPC), which belong to the class of lysophospholipids, are considered important components of lipid droplet monolayers in mammalian cells. Arachidonic acid has been considered a key unsaturated fatty acid associated with various diseases including diarrhea (Burns et al., 2018; Hua et al., 2020; Naito et al., 2015; Shi et al., 2023). Herein, the content of arachidonic acid (peroxide free) was associated with diarrhea traits and significantly increased in the first 4 weeks of age in calves. Correlation analysis revealed a positive correlation between elevated fecal metabolites and harmful intestinal microorganisms, along with a negative correlation with growth performance.

The correlation between arachidonic acid (peroxide free) and growth performance traits in calves, such as average daily gain, chest circumference, and hip height showed a significant negative correlation. This suggests that the increase in arachidonic acid (peroxide free) may be a contributing factor to the stunted growth observed in diarrheic calves. Further analysis revealed a positive correlation between arachidonic acid (peroxide free) and harmful fecal metabolites and microorganisms such as *GCA_900066575,* Gammaproteobacteria, and Proteobacteria, while showing a significant negative correlation with beneficial bacteria *Lachnoclostridium_sp_YL32* and *Clostridium_scindens*. Studies have found that *Escherichia-shigella* was significantly correlated with metabolites involved in the metabolism of arachidonic acid and *α*-linolenic acid such as phosphatidylcholine and lecithin (Yan et al., 2022). Our findings also revealed that *Escherichia-shigella* was positively correlated with arachidonic acid (peroxide free). It can be speculated that arachidonic acid (peroxide free) is associated with an increase in Gammaproteobacteria and Proteobacteria, which results in the production of pro-inflammatory factors and may have stunted the growth and development of calves.

Herein, 7-keto-8-aminopelargonic acid was found in D1W diarrheic calves, which results in the production of demercaptobiotin after an ATP-dependent carboxylation reaction. Biotin is a cofactor of carboxylase, and is produced by various gut bacteria, being considered essential for glucose, amino acid, and fatty acid metabolism (Bi et al., 2016; Yoshii et al., 2019). It has also anti-inflammatory properties by inhibiting nuclear factor kappa-B activity (Elahi et al., 2018). Free biotin is essential for the growth and survival of the microbiota, and biotin deficiency will affect the composition of the intestinal microbiota in calves, leading to dysbiosis (Hayashi et al., 2017), which may be the reason for the significant decrease in metabolites related to biotin metabolism, TCA cycle, and lipopolysaccharide biosynthesis in diarrheic calves. Finally, a strong correlation was found between the excessive abundance of *Fusobacterium* and the loss of certain potentially beneficial bacterial genera (Yoshii et al., 2019), which might play a role that biotin deficiency contributes to intestinal microbiota disruption.

## 5 Conclusion

In the present study, it was found that diarrhea significantly reduced average daily gain and BW in calves during the first 4 weeks after birth. Significant changes were found in fecal microbiota composition and metabolite profiles in diarrheic calves, characterized by an increased abundance of intestinal pathogenic bacteria and imbalanced metabolite levels. Key bacteria such as *Enterococcus_cecorum*, *Lachnospiraceae_UCG-004*, *Tyzzerella*, *Clostridium_butyricum Anaerotignum_lactatifermentans*, and *Escherichia-shigella* were identified as primary contributors to diarrhea in calves. The main underlying mechanism leading to reduced growth and development in diarrheic calves could be associated to an increase in certain metabolites such as arachidonic acid (peroxide free), fatty acids, and glycerophospholipids. Conversely, beneficial bacteria such as *Lachnoclostridium_sp_YL32*, *Bifidobacterium_pseudocatenulatum_DSM_20438_=_JCM_1200_=_LMG_10505*, *UBA1819*, and *Clostridium_scindens* could potentially aid in preventing and controlling diarrhea in calves. These findings provide potential biomarkers for the targeted regulation of intestinal microbiota and metabolites, offering insights into strategies for preventing and controlling diarrhea in calves.

## Data Availability

The datasets presented in this study can be found in online repositories. The names of the repository/repositories and accession number(s) can be found in the article/Supplementary material.

## Footnotes

^1^ https://www.omicsmart.com
