# Supplementary material for: 16S rRNA and metabolomics reveal the key microbes and key metabolites that regulate diarrhea in Holstein male calves
Source: Front Microbiol. 2025 Jan 15;15:1521719. doi: 10.3389/fmicb.2024.1521719 (PMC11778179; doi:10.3389/fmicb.2024.1521719)
Supplement: Supplementary file 1 [file Supplementary_file_1.docx]

**Supplementary Material 2**

**1 Supplementary Fig. S1.** Significant differences in rectal flora between healthy and diarrheal calves.

At the class level, compared with H1W, there was no significant change in phylum of fecal flora of D1W calves (*P* > 0.05). Compared with H2W, *Gammaproteobacteria* in fecal flora of D2W calves was significantly higher (Figure S1A, *P* < 0.05). Compared with H3W, *Gammaproteobacteria* in fecal flora of D3W calves was also significantly higher (Figure S1B, *P* < 0.05). Compared with H4W, there were no significant changes in the outline of fecal flora of D4W calves (*P* > 0.05). At the family level, Actinomycetaceae in the fecal flora of D1W calves was significantly higher than that of H1W calves (Figure S1C, *P* < 0.05). Compared with H2W, Enterobacteriaceae in the fecal flora of D2W calves was significantly higher (Figure S1D, *P* < 0.05). Compared with H3W, there were no significant changes in the fecal flora of D3W calves (*P* > 0.05). Compared with H4W, the fecal flora of D4W was significantly higher in *Oscillospiraceae*, and significantly lower in *Eubacterium_coprostanoligenes_group* and *Eubacteriaceae* (Figure S1E, *P* < 0.05).


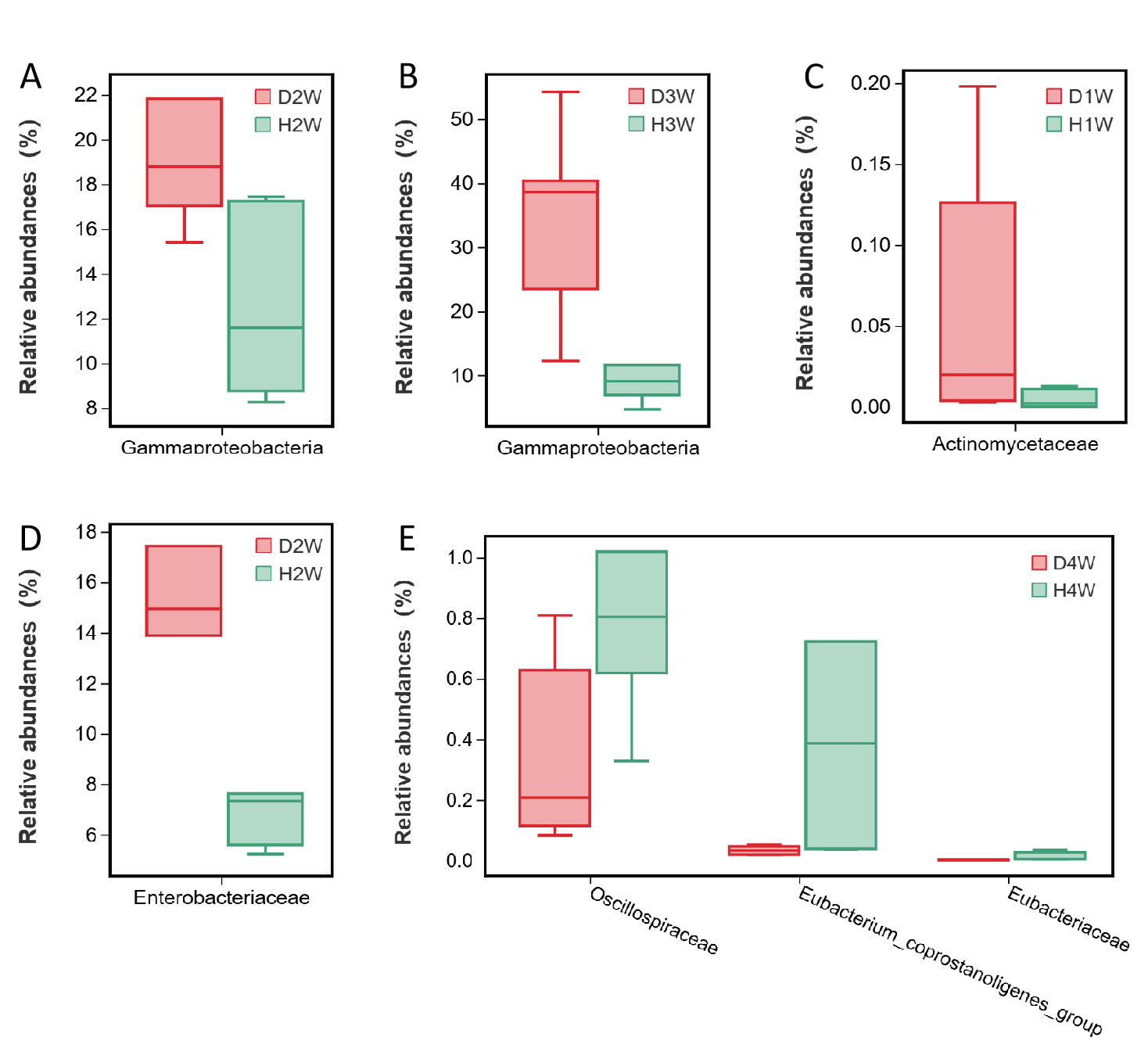


**Fig. S1.** Significant differences in rectal flora between healthy and diarrheal calves. Wilcoxon rank sum test of D2W and H2W fecal flora of calves at Class level (A). Wilcoxon rank sum test of D3W and H3W fecal flora of calves at Class level (B). Wilcoxon rank sum test of D1W and H1W fecal flora of calves at family level (C). Wilcoxon rank sum test of D2W and H2W fecal flora of calves at family level (D). Wilcoxon rank sum test of D4W and H4W fecal flora of calves at family level (E).

**2 Supplementary Fig. S2:** Analysis of Fecal Metabolism Characterization of Calves With Diarrhea


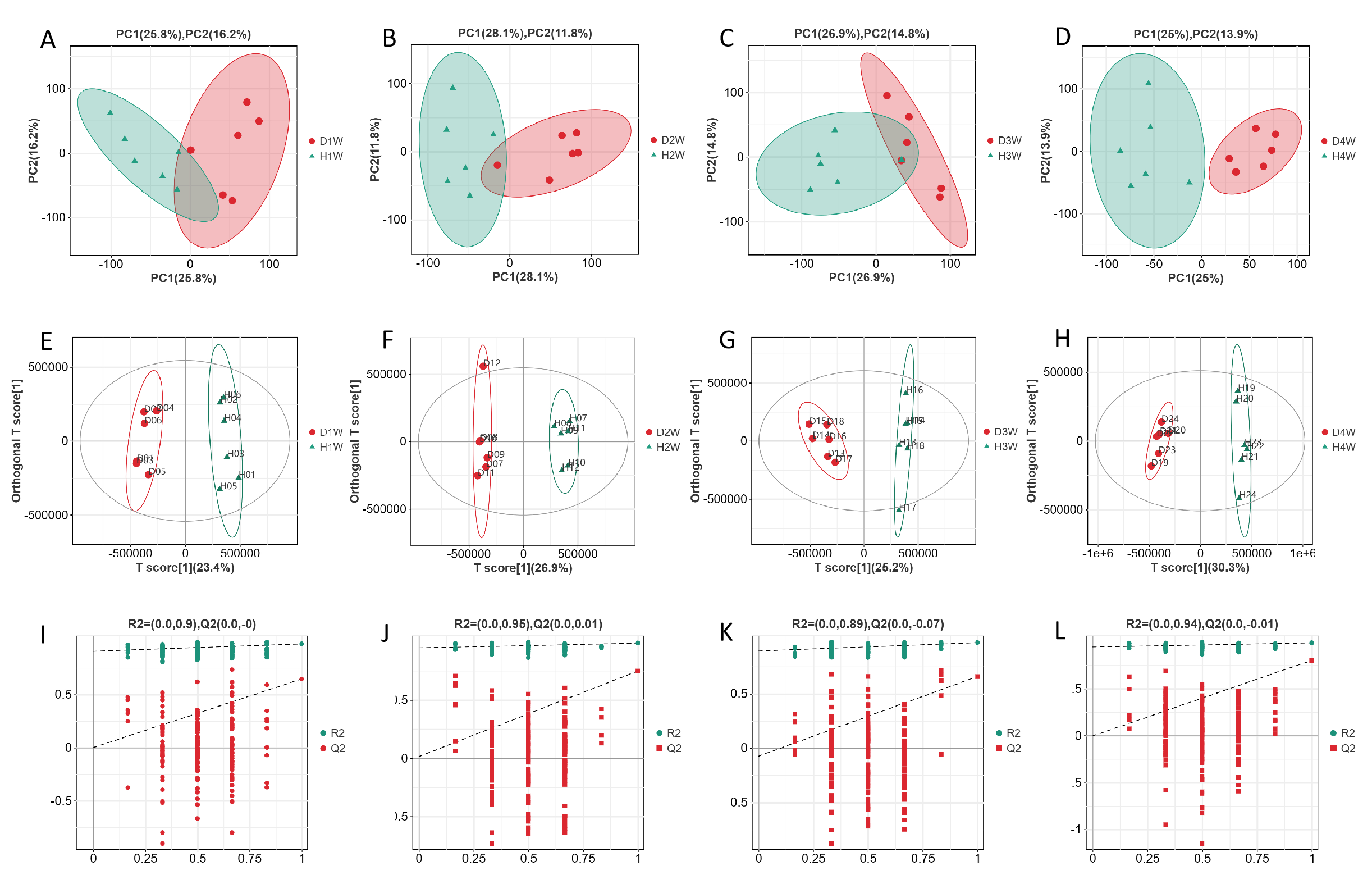


**Figure S2.** Analysis of fecal metabolic characteristics of healthy calves and diarrheal calves. PCA of fecal metabolites in D1W and H1W calves (A). PCA of fecal metabolites in D2W and H2W calves (B). PCA of fecal metabolites in D3W and H3W calves (C). PCA of fecal metabolites in D4W and H4W calves (D). OPLS-DA score plots of fecal metabolites in D1W and H1W calves (E). OPLS-DA score plots of fecal metabolites in D2W and H2W calves (F). OPLS-DA scoring charts of fecal metabolites in D3W and H3W calves (G). OPLS-DA scoring charts of fecal metabolites in D4W and H4W calves (H). Alignment test of OPLS-DA models for fecal metabolites in D1W and H1W calves (I). Alignment test of OPLS-DA models for fecal metabolites in D2W and H2W calves (J). Alignment test of OPLS-DA models for fecal metabolites in D3W and H3W calves (K). Alignment test of OPLS-DA models for fecl metabolites in D4W and H4W calves (L).
