# Supplementary material for: 16S rRNA and metabolomics reveal the key microbes and key metabolites that regulate diarrhea in Holstein male calves
Source: Front Microbiol. 2025 Jan 15;15:1521719. doi: 10.3389/fmicb.2024.1521719 (PMC11778179; doi:10.3389/fmicb.2024.1521719)
Supplement: Supplementary file 2 [file Data_Sheet_1.docx]

**Supplementary Material 1**

**1 Supplementary Table S1**

Analysis of milk composition in normal milk

| Milk composition | Value |
| --- | --- |
| Fat（F, %） | 4.19±0.19 |
| Non-fat milk solids（S, %） | 9.23±0.07 |
| Density of milk（D） | 30.65±0.27 |
| Protein（P, %） | 3.49±0.03 |
| Freezing point（FP） | 60.13±0.39 |
| Temperature（T, ℃） | 27.90±0.66 |
| Titratable acidity（TA, Th） | 19.75±0.18 |
| Conductivity（Z） | 4.05±0.03 |
| pH value（PH） | 6.54±0.01 |
| Water content added（W, %） | 0.00±0.00 |

**2 Supplementary Table S2**

Nutrient composition analysis of concentrate supplements

| Nutrient composition of granular materials | Value |
| --- | --- |
| Moisture（%） | ≤14.00 |
| Crude protein（CP, %） | ≥20.0 |
| Crude fiber（CF, %） | ＜10.0 |
| Crude ash（%） | ≤12.0 |
| Calcium（Ca, %） | 0.30-3.00 |
| Total phosphorus（TP, %） | ≥0.30 |
| Sodium oxide（NaO, %） | 0.30-2.50 |
| Lysine（Lys, %） | ≥0.50 |

**3 Supplementary Table S3**

Calf body length ruler index measurement

| Index | Measuring point |
| --- | --- |
| withers height | From the highest point of the first vertical to the ground height |
| chest circumference | Make a vertical line at the posterior edge of the scapula, measure it with a tape measure for 1 week, and can slide up and down the index and middle fingers |
| height at hip cross | The center of the waist corner (the cross) is vertical to the height of the ground |
| body length | Distance from the shoulder to the scium |
| hip height | The height of the sciatic nodule to the ground |
| hip width | The left and right two waist angles are the maximum width |
| hucklebone width | The width of the outermost tuberosity of the left and right sciatic nodules |
| rump length | Straight distance from the leading edge of the waist angle to the posterior edge of the jiri end |
| circumference of cannon bone | The circumference diameter of 1/3 on the neck of the forelimb is generally measured at the finest part of the anterior tube |
